# CHCHD2 mutant mice link mitochondrial deficits to PD pathophysiology

**DOI:** 10.1126/sciadv.adu0726

**Published:** 2025-11-14

**Authors:** Szu-Chi Liao, Kohei Kano, Sadhna Phanse, Mai Nguyen, Elyssa Margolis, YuHong Fu, Jonathan X. Meng, Mohamed Taha Moutaoufik, Zac Chatterton, Tatiana Saccon, Kirsten Broderick, Hiroyuki Aoki, Jeffrey Simms, Felicia Xaveria Suteja, Yoshitaka Sei, Eric J. Huang, Kevin McAvoy, Giovanni Manfredi, Glenda Halliday, Mohan Babu, Ken Nakamura

**Affiliations:** ^1^ Gladstone Institute of Neurological Disease, Gladstone Institutes, San Francisco, CA, USA.; ^2^ Department of Nutritional Sciences and Toxicology, University of California, Berkeley, Berkeley, CA, USA.; ^3^ Endocrinology Graduate Program, University of California, Berkeley, Berkeley, CA, USA.; ^4^ Aligning Science Across Parkinson’s (ASAP) Collaborative Research Network, Chevy Chase, MD, USA.; ^5^ Department of Biochemistry, University of Regina, Regina, Saskatchewan, Canada.; ^6^ Weill Institute for Neurosciences, Department of Neurology, and Kavli Institute for Fundamental Neuroscience, University of California San Francisco, San Francisco, CA, USA.; ^7^ Neuroscience Graduate Program, University of California San Francisco, San Francisco, CA, USA.; ^8^ Brain and Mind Centre and Faculty of Medicine and Health School of Medical Sciences, University of Sydney, Sydney, Australia.; ^9^ Faculty of Medical Sciences, UM6P Hospitals, Mohammed VI Polytechnic University, Ben Guerir, Morocco.; ^10^ Department of Pathology, University of California San Francisco, San Francisco, CA, USA.; ^11^ Biomedical Sciences Graduate Program, University of California San Francisco, San Francisco, CA, USA.; ^12^ Feil Family Brain and Mind Research Institute, Weill Cornell Medicine, New York, NY, USA.

## Abstract

Mitochondrial dysfunction is a hallmark of Parkinson’s disease (PD), but the mechanisms by which it drives autosomal dominant and idiopathic forms of PD remain unclear. To investigate this, we generated and performed a comprehensive phenotypic analysis of a knock-in mouse model carrying the T61I mutation in the mitochondrial protein CHCHD2 (coiled-coil–helix–coiled-coil–helix domain–containing 2), which causes late-onset symptoms indistinguishable from idiopathic PD. We observed pronounced mitochondrial disruption in substantia nigra dopaminergic neurons, including distorted ultrastructure and CHCHD2 aggregation, as well as disrupted mitochondrial protein-protein interactions in brain lysates. These abnormalities were associated with a whole-body metabolic shift toward glycolysis, elevated mitochondrial reactive oxygen species (ROS), and progressive accumulation of aggregated α-synuclein. In idiopathic PD, *CHCHD2* gene expression also correlated with α-synuclein levels in vulnerable dopaminergic neurons, and CHCHD2 protein accumulated in early Lewy aggregates. These findings delineate a pathogenic cascade in which CHCHD2 accumulation impairs mitochondrial respiration and increases ROS production, driving α-synuclein aggregation and neurodegeneration.

## Teaser

A CHCHD2 mouse model of Parkinson’s disease exhibits mitochondrial disruption with a metabolic shift toward glycolysis.

## INTRODUCTION

Mitochondria have long been hypothesized to play a central role in the pathophysiology of Parkinson’s disease (PD). Sporadic PD has been repeatedly associated with decreased respiratory chain activity, especially mitochondrial complex I in dopaminergic (DA) neurons in the substantia nigra pars compacta (SNc) (*1*–*3*). Aging is also the greatest risk factor for PD, and mitochondrial DNA mutations accumulate at a faster rate in SNc dopaminergic (DA) neurons in PD than in normal aging (*4*, *5*). Moreover, neurotoxin data suggest that SNc DA neurons are preferentially vulnerable to mitochondrial complex I dysfunction, as exposure to the mitochondrial complex I inhibitors rotenone and 1-methyl-4-phenyl-1,2,3,6-tetrahydropyridine (MPTP) either predispose to PD (rotenone) or cause a parkinsonian syndrome (MPTP) (*6*–*8*).

Although the neuropathologic and neurotoxin data implicate mitochondria in PD pathophysiology, they do not establish that changes in mitochondria actually cause PD. The most direct evidence for a causal link between mitochondrial dysfunction and PD comes from mutations in the mitochondrial protein PINK1 and the mitochondria-associated protein Parkin, which cause familial forms of PD (*9*). Moreover, the roles of PINK1 and Parkin in mitochondrial turnover, biogenesis, motility, and bioenergetics suggest potential mechanisms by which mitochondria may cause PD (*10*–*16*). However, the absence of robust mitochondrial or other deficits in *PINK1* and *Parkin* mutant or knockout (KO) mice (*17*–*20*), as well as the identification of nonmitochondrial functions of these proteins (*21*–*25*), leaves uncertainty about the role of mitochondria in PINK1- and Parkin-mediated PD. In addition, most patients with *Parkin* mutations, the most frequent cause of autosomal recessive PD, do not have Lewy body pathology (*26*). This has led many investigators to hypothesize that autosomal recessive forms of PD are distinct disease subtypes, differing mechanistically from autosomal dominant and sporadic forms of PD that more closely resemble each other (*27*).

As such, the discovery that mutations in the mitochondrial intermembrane space protein CHCHD2 (coiled-coil–helix–coiled-coil–helix domain–containing 2) cause an autosomal dominant form of late-onset PD that closely resembles sporadic PD (*28*)—including widespread α-synuclein deposition (*29*)—proves that mitochondrial dysfunction can cause autosomal dominant PD, and strongly implicates it as a causal factor in at least a subset of idiopathic PD. It also provides an important opportunity to learn how mitochondrial dysfunction can cause PD.

However, understanding how mutations in CHCHD2 promote PD has proven challenging. Although CHCHD2 may in some way support respiration (*30*–*32*), its physiological functions remain unclear, especially in vivo. Moreover, mice lacking CHCHD2 exhibit only subtle phenotypes (*33*, *34*), suggesting that CHCHD2 mutations may produce toxicity through gain of function. This possibility is supported by recently described mouse models with mutant CHCHD2 (*35*–*37*) and by the finding that mutations in CHCHD10, CHCHD2’s paralog and binding partner, appear to produce toxicity through gain of function (*38*). However, how exactly mutant CHCHD2 affects mitochondria and their functions in vivo and ultimately leads to neuronal dysfunction and preferential degeneration of nigrostriatal dopamine neurons remains unclear. To address this gap, we conducted a comprehensive analysis of the CHCHD2 T61I phenotype using a mouse model we generated and extended key observations to brain tissue from patients with sporadic PD.

## RESULTS

### Generation of CHCHD2 T61I mice with CRISPR genome editing

**Fig. 1.**
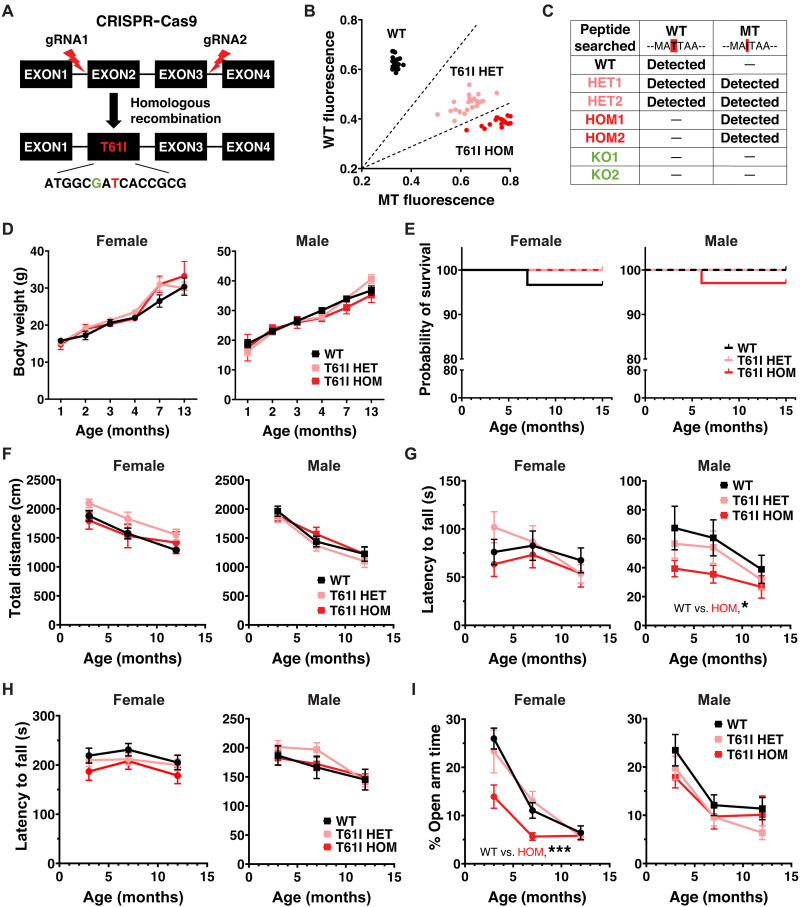
CHCHD2 T61I mice exhibit normal survival and subtle motor deficits. (**A**) Insertion of CHCHD2 T61I point mutation by CRISPR-Cas9 gene editing and homologous recombination. (**B**) CHCHD2 T61I mouse single-nucleotide polymorphism genotyping by qPCR. Three clusters were autogenerated by allelic discrimination in the analysis software, including WT (black), T61I HET (pink), and T61I HOM (red). (**C**) WT peptide (MAQMA**T**TAAGVAVGSAVGHTLGHAITGGFSGGGSAEPAKPDITYQEPQGAQL) and mutant peptide (MAQMA**I**TAAGVAVGSAVGHTLGHAITGGF) were searched in brain lysates of seven mice of different genotypes (one WT, two T61I HET, two T61I HOM, and two CHCHD2 KO). WT peptide was found in WT and HET mice, while mutant was found in HET and HOM mice. Neither peptide was found in KO mice. (**D**) CHCHD2 T61I mice showed similar body weights compared with WT through 13 months of age. *N* = 5 to 9 mice per genotype per gender. (**E**) T61I mice showed normal survival compared with WT in Kaplan-Meier survival curve through 16 months of age. Left, females (*n* = 30 WT, 18 HET, and 17 HOM); right, males (*n* = 21 WT, 20 HET, and 34 HOM). (**F**) Total movements of T61I mice were normal in the open field test. (**G**) Male T61I HOM mice showed a significant decrease in latency to fall in inverted grid hang. (**H**) Latency to fall in the accelerating rotarod test was not changed in T61I mice. (**I**) Female HOM showed significantly lower time spent in the open arms. In all behavioral studies [(F) to (I)], *n* = 16 WT, 16 HET, and 13 HOM (female), or 14 WT, 18 HET, and 17 HOM (male). In all panels, data represent mean ± SEM. *P < 0.05, ***P < 0.001 by linear mixed-effects analysis.

CHCHD2 p.T61I mice were generated using CRISPR-Cas9 genome editing (Fig. 1A). Two single guide RNAs (sgRNAs) were used to delete exons 2 and 3, and a targeting donor vector was used to deliver the cytosine-to-thymine mutation, resulting in the codon change from threonine to isoleucine. This approach allowed us to specifically target *CHCHD2* without affecting *Zbed5*, which shares high sequence identity with *CHCHD2* in rodents (*35*). A silent cytosine-to-guanine mutation 2 nucleotide immediately upstream was also introduced for genotyping purposes.

Because of the signal overlap from *Zbed5* in Sanger sequencing, we used quantitative polymerase chain reaction (qPCR) to perform single-nucleotide polymorphism (SNP) genotyping of the mice (Fig. 1B). Different genotypes were distinguished using wild-type (WT) and mutant probes based on clustering. As orthogonal confirmation, liquid chromatography with tandem mass spectrometry (LC-MS/MS) of total brain protein lysates was used to identify WT and mutant peptides (Fig. 1C).

### CHCHD2 T61I mice show normal life span and subtle motor deficits

CHCHD2 p.T61I mice were born in normal Mendelian proportions [WT: 26.9%, HET (heterozygous): 42.6%, HOM (homozygous): 30.6%, *n* = 108] and had similar body weights to littermate controls through 13 months of age (Fig. 1D). Survival was also unchanged through 16 months (Fig. 1E). A small subset of mice tracked for 23 months also failed to show any effects on survival (fig. S1A).

We next assessed whether the CHCHD2 T61I mutation disrupts motor function. We studied both heterozygous T61I mice, which model the heterozygous T61I expression observed in PD, and homozygous mutant mice, which we hypothesized would have stronger phenotypes. T61I homozygous and heterozygous mutation had no overall effects on total movement in open field testing across the three time points (3, 7, or 12 months) (Fig. 1F). Repeated open field testing with the automated tracking system, EthoVision XT, on a small cohort of 23-month-old male mice also failed to show differences in total movement or time spent moving (fig. S1, B and C). Although total movement was not affected, male (but not female) homozygous T61I mice exhibited a significantly shorter latency to fall in inverted grid hang test (Fig. 1G). No significant differences were found in rotarod testing (Fig. 1H). In the elevated plus maze, female T61I homozygous mutants showed a trend-level decrease in distance traveled (fig. S1D). Overall, these findings are consistent with subtle motor deficits in the T61I mice, with no evidence of progression with older age. In contrast to homozygotes, analysis of the motor function of CHCHD2 T61I heterozygous mice revealed only a small difference in distance traveled on elevated plus maze versus controls (fig. S1D), with no other differences across the behavioral assessments (Fig. 1, F to H, and fig. S1, B to C).

Mice with the T61I mutation also showed evidence of nonmotor changes. In elevated plus maze testing, female homozygous T61I mice spent considerably less time in the open arms, exhibited a reduced open arm/total distance percentage, and entered the open arms fewer times, aligning with increased anxiety (Fig. 1I and fig. S1D).

### CHCHD2 T61I disrupts dopamine autoreceptor function but does not cause significant degeneration of mouse SNc DA neurons

**Fig. 2.**
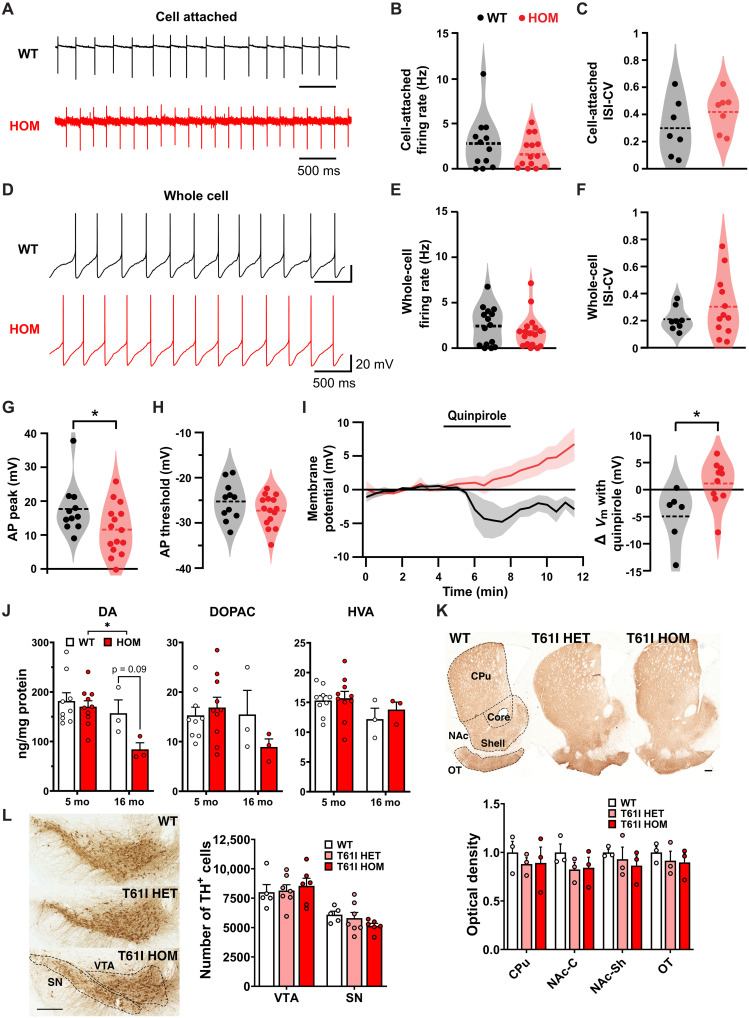
CHCHD2 T61I point mutation disrupts DA autoreceptor function without DA neuron degeneration. Recordings were made blind to genotype. (**A** to **C**) SNc DA neurons from CHCHD2 T61I and control mice fired spontaneously in acute brain slices in cell attached recording configuration. (A) Example recording traces. (B) Spontaneous firing rates in DA neurons in cell-attached mode were similar. (C) Regularity of spontaneous firing evaluated by the coefficient of variation of 100 consecutive interspike intervals (ISI-CV). (**D**) Example of whole-cell current-clamp (*I* = 0 pA) recordings. (**E** and **F**) Firing rates and ISI-CVs were similar in whole-cell recording configuration. (**G** and **H**) Spontaneous action potential (AP) waveform, AP peak (G) and threshold (H). (**I**) The D2R selective agonist quinpirole (1 μM) caused hyperpolarization in most WT but not T61I mutant neurons. Left: time course response averages; right: summary. In (A) to (I), *N* = 5 WT and 4 HOM male mice at 23 months; each dot is a neuron. (**J**) HPLC of dorsal CPu punches at 5 or 16 months showed lower DA level in T61I HOM mice. HVA, homovanillic acid; DOPAC, 3,4-dihydroxyphenylacetic acid. *N* = 9 to 10 (5 months) or 3 (16 months) mice per genotype. (**K** and **L**) Representative images of TH immunoreactivity in striatal (K) or midbrain sections (L) of T61I mice at 16 months. There was no difference in either TH optical density in the striatum or stereology cell counts in the midbrain. Subregions are indicated with dotted lines. CPu, caudate putamen; NAc, nucleus accumbens; OT, olfactory tubercle. *N* = 3 mice per genotype, four sections per animal (K) or five to seven mice per genotype (L). Scale bar: 300 μm. In all panels, data represent mean ± SEM or with kernel density estimations. *P < 0.05 by *t* test (parametric) or permutation (nonparametric) analysis [(A) to (I)] or two-way ANOVA with Sidak’s (J) or Tukey’s [(K) and (L)] post hoc test.

We next assessed whether the T61I mutation affects the somatodendritic physiological health of SNc DA neurons. Ex vivo whole-cell recordings from 23-month-old male mice showed that basic physiological properties of SNc DA neurons in homozygous T61I mice were similar to those in control SNc neurons. As expected, most DA neurons fired spontaneously in a regular, pacemaker fashion in both cell-attached and whole-cell configuration (Fig. 2, A to F). On average, action potentials in DA neurons from the T61I mice had a less depolarized peak compared to controls (Fig. 2G). A parallel but not significant pattern was observed in action potential threshold (Fig. 2H). There was no difference in action potential durations (fig. S2A). A small population of DA neurons from the T61I mice showed less regularity of firing (greater coefficient of variation of interspike intervals) compared to WT DA neurons (Fig. 2F); however, overall, this measurement was not significantly different between groups. *I*_h_ magnitude and input resistance distributions were also similar between SNc DA neurons from WT and T61I mice (fig. S2, B and C).

There was, however, one major neurophysiological alteration in DA neuron function in the CHCHD2 T61I mice. Dopamine release in the SNc inhibits further release by activating dopamine D2 receptors (D2Rs) on the dopamine-producing neurons (*39*–*41*). We found that in CHCHD2 T61I mice, treatment with a D2R agonist failed to elicit D2R hyperpolarization in SNc DA neurons (Fig. 2I). The lack of D2R agonist-induced hyperpolarization may be due to one or more of the following factors: down-regulation of D2Rs, down-regulation of the G protein receptor gated inwardly rectifying K^+^ channels responsible for the hyperpolarization response, or D2R coupling changing to an alternative signaling pathway. This loss of autoinhibition could enhance DA neuron firing activity in vivo in certain behavioral conditions.

To determine whether CHCHD2 T61I affects dopamine metabolism, we assessed dopamine levels in the dorsal striatum, the primary target of dopaminergic projections from the SNc. T61I homozygous mice showed lower levels of DA when considered across 5 and 16 months (Fig. 2J), suggesting disrupted dopamine signaling. Other monoamines and monoamine metabolites were also detected but not significantly different between groups (Fig. 2J and fig. S2D).

We next examined whether the T61I mutation leads to DA neuron degeneration. First, to assess the integrity of DA neuron terminals, we quantified tyrosine hydroxylase (TH) expression in the striatum using 3′3-diaminobenzidine (DAB) staining. There was no significant effect of either heterozygous or homozygous T61I mutation on TH optical density in the striatum of 16-month-old mice (Fig. 2K). A second cohort of 23-month-old male mice also showed no difference between the T61I and WT mice (fig. S2E). We then performed stereology to quantify the number of DA neurons in the midbrain and similarly found no significant loss of DA neurons in either the substantia nigra (SN) or ventral tegmental area (VTA) (Fig. 2L).

### Preferential accumulation of CHCHD2/10 and disrupted mitochondrial ultrastructure in SNc DA neurons

**Fig. 3.**
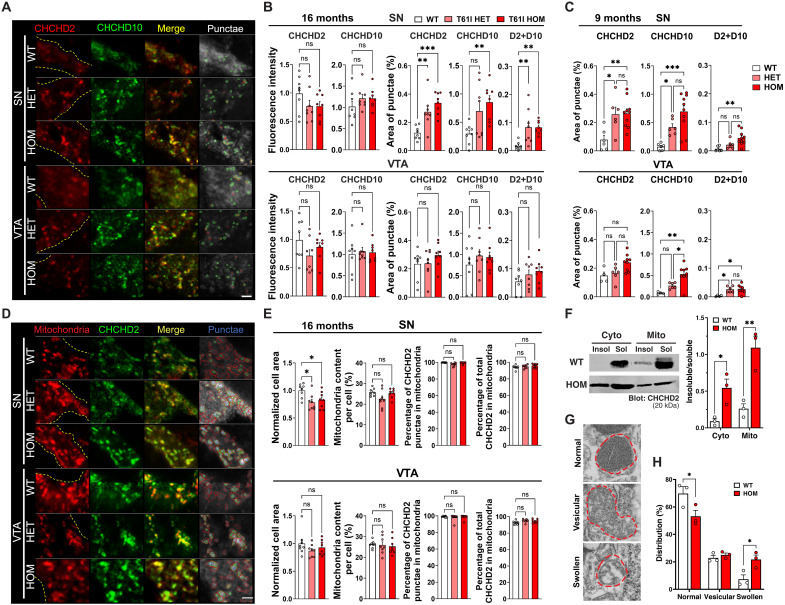
T61I point mutation increases accumulation of CHCHD2 and CHCHD10 in mitochondria preferentially in SN DA neurons. (**A**) Representative fluorescence images of CHCHD2 (red), CHCHD10 (green), and merge in subregions in the midbrain of T61I mice at 16 months. Punctae were semiautomatically annotated by CellProfiler. (**B** and **C**) Quantification of CHCHD2 and CHCHD10 intensity (B only), punctae area, and punctae colocalization at 16 (B) or 9 (C) months. (**D**) Representative fluorescence images of mitochondria (PDH, red), CHCHD2 (green), and merge at 16 months. CHCHD2 punctae in blue. (**E**) Quantification of cell area, mitochondrial content, CHCHD2 punctae in mitochondria, and percent of CHCHD2 in mitochondria. In (A) to (E), *n* = 6 to 8 TH^+^ cells (six fields per region per mouse, four mice per genotype at 16 months or three to five mice per genotype at 9 months). Mitochondrial content and punctae area were normalized to cell area. In (A) and (D), the cell body is outlined with dotted lines based on TH signal. Examiner was blinded to genotype. Scale bar, 1 μm. (**F**) Western blot revealed increased insolubility of T61I CHCHD2 in both mitochondrial and cytosolic brain lysate fractions. *N* = 2 mice per genotype at 16 months from three independent experiments. (**G**) Electron microscopy images of normal, vesicular, or swollen mitochondria in SN DA neurons (19,000× magnification) labeled with primary TH antibody followed by secondary gold particle–conjugated antibody. (**H**) T61I HOM mice had a higher percentage of swollen mitochondria. *N* = 71 to 189 mitochondria per mouse, three mice per genotype. In all panels, data represent mean ± SEM. *P < 0.05, **P < 0.01, ***P < 0.001 by one-way ANOVA with Tukey’s post hoc test [(B), (C), and (E)] or two-way ANOVA with Sidak’s post hoc test [(F) and (H)].

CHCHD2 forms homodimers and heterodimers with CHCHD10 in mitochondria under physiological conditions (*30*). Moreover, homodimers and heterodimers containing T61I CHCHD2 are prone to aggregate (*29*, *30*). To assess the effects of T61I mutation on the expression, subcellular distribution, and spatial organization of CHCHD2, we used super-resolution microscopy on 16-month-old mice. First, we found that T61I did not change the level of CHCHD2 expression in either SN or VTA DA neurons (Fig. 3, A and B). Similarly, the level of CHCHD10 expression was comparable between neurons of all genotypes. However, both heterozygous and homozygous T61I-expressing DA neurons had markedly increased size of CHCHD2 and CHCHD10 punctae, specifically in the SN, and not the VTA. Here, punctae are defined as foci of CHCHD2 or CHCHD10 immunofluorescence that could represent either CHCHD2 and CHCHD10 aggregation, or focal areas of increased CHCHD2 and CHCHD10 levels in mitochondria. Notably, CHCHD2 and CHCHD10 punctae were also increased in the SN at an earlier time point, 9 months, although the overall punctae level was lower than at 16 months (Fig. 3C), suggesting that protein buildup occurs in an age-dependent manner.

Because both CHCHD2 and CHCHD10 are predominantly localized to the mitochondrial intermembrane space (*42*), we next assessed whether T61I CHCHD2 affects the mitochondrial content in either SNc or VTA DA neurons at 16 months. We found that T61 CHCHD2 heterozygous and homozygous DA neurons had similar mitochondrial content to controls, based on immunofluorescence against the inner mitochondrial marker pyruvate dehydrogenase (PDH) (Fig. 3, D and E). In addition, almost all of the T61 CHCHD2 and WT CHCHD2 colocalized with mitochondria (Fig. 3E).

To gain insight into whether the accumulation of CHCHD2 punctae reflects a change in CHCHD2 protein solubility, for instance, due to CHCHD2 aggregation, we performed Western blots on cytosolic and mitochondrial fractions (*43*) from total brain lysates of 16-month-old mice (Fig. 3F). T61I CHCHD2 homozygous mice had increased levels of insoluble versus soluble CHCHD2 in both cytosolic and mitochondrial fractions, with the latter being more abundant, suggesting greater aggregation of T61I CHCHD2 within mitochondria.

The accumulation of T61I CHCHD2 mutant and CHCHD10 within mitochondria suggests potential disruption of mitochondria. To address whether mitochondrial cristae were affected by protein accumulation, we labeled DA neurons using immunogold staining and assessed the mitochondrial ultrastructure. Following the definition by Eustaquio *et al.* (*44*), we categorized mitochondrial morphology into three groups: normal, vesicular, and swollen (Fig. 3G). Compared with WT mice, T61I CHCHD2 homozygous mice showed a significant decrease in normal form and a significant increase in swollen form of mitochondria in SN DA neurons (Fig. 3H and fig. S2F), indicating mitochondrial damage by CHCHD2 T61I mutation.

### CHCHD2 expression levels are altered in vulnerable DA neurons in sporadic PD

**Fig. 4.**
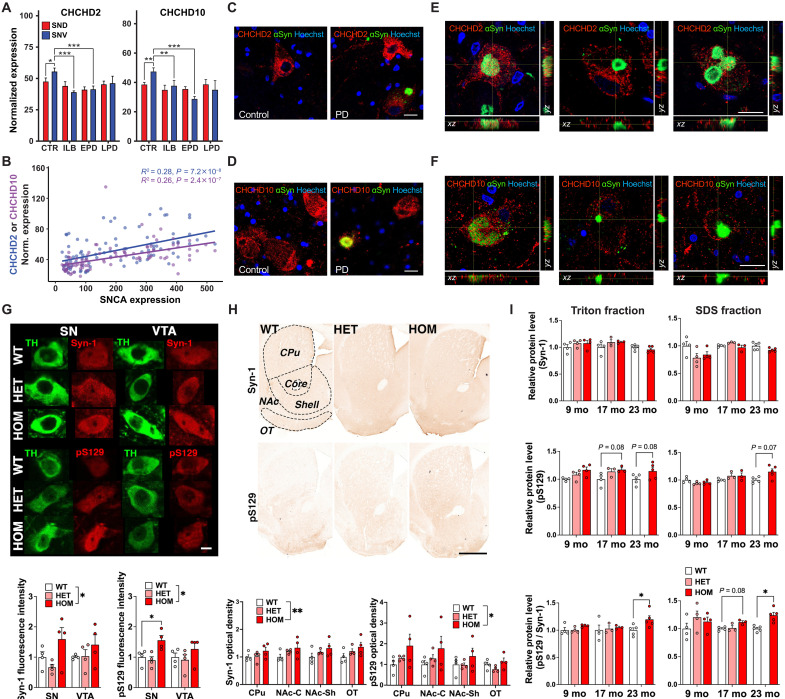
Interrelationship between CHCHD2 and α-synuclein in PD and PD mouse models. (**A**) GeoMx transcriptomics showed significant down-regulation of *CHCHD2* and *D10* in DA neurons of SNV (ventral tier of SN) but not SND (dorsal) of ILB and EPD cases. CTR, control; ILB, incidental Lewy body; EPD or LPD, PD with early or late Braak stage pathology. (**B**) Positive linear associations between levels of *CHCHD2* or *D10* and *SNCA* in the SN. (**C** and **D**) CHCHD2 (C) or D10 (D) localization in control and PD SN DA neurons. (**E** and **F**) Location of CHCHD2 (E) or D10 (F) in different staged αSyn aggregations. Scale bars, 50 μm [(C) and (D)] or 20 μm [(E) and (F)]. Numbers of human samples [(A) to (F)] in table S1. (**G** and **H**) Representative images and quantification of total α-synuclein (Syn-1) or phosphorylated α-synuclein (pS129) immunoreactivity in midbrain (G) or striatal sections (H) of 16-month-old mice. Syn-1 and pS129 immunoreactivities increased in HOM mice when considered across SN and VTA (G) or four subregions in the striatum (H), although differences within some individual regions did not reach significance. Dotted lines delineate subregions in the striatum. Scale bars, 10 μm (G) or 300 μm (H). *N* = 4 sections per mouse, four mice per genotype. (**I**) Quantification of α-synuclein in soluble (Triton X-100) and insoluble (SDS) fractions of frontal cortex lysates. pS129 and pS129/total α-synuclein ratio increased progressively in HOM mice from 9 to 23 months. Protein levels were normalized to total protein determined by Coomassie brilliant blue staining (fig. S3G). *N* = 3 to 5 mice per genotype at each time point (except two HET in SDS fraction at 17 months). mo, months. In all panels, data represent mean ± SEM. *P < 0.05, **P < 0.01, ***P < 0.001 by LIMMA contrasts (A) or two-way ANOVA with Tukey’s post hoc test [(G) to (I)].

The accumulation of aggregated CHCHD2 in the mitochondria of SNc DA neurons from T61I CHCHD2 mice, as well as the associated disruption of their mitochondrial ultrastructure, suggests that changes in CHCHD2 conformation could contribute to disease pathophysiology in CHCHD2 PD. It also raises the question of whether CHCHD2 could also accumulate and contribute to degeneration in sporadic PD. To assess this, we compared *CHCHD2* gene expression in DA neurons in midbrain samples from patients with sporadic PD and age-matched human controls, using spatial genomics (GeoMx WTA) (Fig. 4A and table S1). In controls, transcriptomic levels of both *CHCHD2* and *CHCHD10* were higher in the more vulnerable DA neurons in the ventral tier of the SN (SNV) than in the more resilient DA neurons in the dorsal tier (SND) (LIMMA adjusted *P* value <2.2 × 10^−9^) (Fig. 4A). As both *CHCHD2* and *CHCHD10* showed positive correlations with *OXPHOS* genes (fig. S3A), this difference could result from a relatively high mitochondrial mass in the SNV compared to the SND (fig. S3B). Considering that DA neurons in both mice and humans have increased levels of both CHCHD2 and CHCHD10 (*33*), these findings suggest either that increased CHCHD2 contributes to the preferential vulnerability of SNV DA neurons or that CHCHD2 is protective.

In patients with early PD (EPD) there was a decrease in *CHCHD2* gene expression in DA neurons in the SNV, but not SND (Fig. 4A), raising the possibility that the high *CHCHD2*-expressing SNV DA neurons were lost or that *CHCHD2* expression in these DA neurons decreases as they begin to degenerate. The latter possibility is supported by the finding that *CHCHD2* expression is also decreased in SNV DA neurons from individuals with incidental Lewy bodies (ILBs) and no significant loss of SNV DA neurons. Notably, this relationship disappeared in the late Lewy pathological stage of PD (LPD), when only the most resilient DA neurons remain. The presence of higher *CHCHD2*-expressing DA neurons in late PD might indicate that those DA neurons with lower *CHCHD2* ultimately die; however, further investigation is needed.

### CHCHD2 and α-synuclein display correlated expression and subcellular localization in SNc DA neurons in sporadic PD

The finding of extensive α-synuclein aggregation in a patient with CHCHD2 T61I mutation suggests that mutant CHCHD2 may promote α-synuclein aggregation (*29*). To gain insight into the relationship between mutant T61I CHCHD2 and synuclein, we first examined the relationship between *CHCHD2* and *SNCA* gene expression by spatial transcriptomics (Fig. 4B). As with *CHCHD2*, *SNCA* expression in controls was higher in DA neurons in the most PD-vulnerable ventral tier (SNV mean ± SD, 196 ± 143) than those in the dorsal tier (SND, 137 ± 101, LIMMCA adjusted *P* value 1.9 × 10^−13^). Considering the known pathogenicity of increased WT α-synuclein (*45*), this suggests that increased α-synuclein levels may contribute to the preferential vulnerability of ventral tier DA neurons. When pooling control subjects (Fig. 4B), regression tests indicated positive linear associations between the level of *SNCA* and *CHCHD2* (*R*^2^ = 0.37, Pearson *P* value 3.5 × 10^−6^) as well as between the level of *SNCA* and *CHCHD10* (*R*^2^ = 0.21, Pearson *P* value = 9.9 × 10^−4^) within the ventral tier.

Next, we examined CHCHD2 localization in SNc DA neurons from PD and age-matched controls (Fig. 4, C and D). In PD samples, a fraction of both CHCHD2 and CHCHD10 colocalized with α-synuclein aggregations at all stages (defined in fig. S3C) of Lewy pathology development (Fig. 4, E and F, and fig. S3, D to F).

### Age-dependent α-synuclein aggregation in CHCHD2 T61I mice

Given the above correlations between *CHCHD2* and *SNCA* gene expression in SNc DA neurons and the distribution of CHCHD2 and CHCHD10 in α-synuclein aggregates in PD cases, we next examined whether α-synuclein accumulates and aggregates in the CHCHD2 T61I mutant mice. We first assessed whether the T61I mutation affects α-synuclein levels specifically in midbrain DA neurons by immunofluorescence staining for total α-synuclein and phosphorylated α-synuclein at serine-129 (pS129) (Fig. 4G). Quantification revealed an increase in pS129 levels in homozygous SNc DA neurons. Total α-synuclein levels were also increased overall in homozygote midbrain DA neurons. Moreover, both α-synuclein and pS129 were increased in the striatum of homozygous T61I mice at 16 months by optical density, while changes in heterozygotes did not reach significance (Fig. 4H). Therefore, T61I homozygous mice accumulated increased levels of both total and phosphorylated α-synuclein both in the SNc DA neurons’ cell body and in the striatum. However, further investigation is required to determine whether SNc DA neurons in heterozygous T61I mice also accumulate more α-synuclein at older age, and whether the increase in the striatum reflects changes in midbrain DA axons versus striatal target neurons.

We next investigated the impact of the CHCHD2 T61I mutation on the chronological progression of α-synuclein aggregation by quantifying α-synuclein protein levels in the cerebral cortex of mutant mice using Western blot analysis (Fig. 4I). At 9 months of age, no differences were observed in total α-synuclein or pS129 levels in either the soluble or insoluble fractions. By 17 months, however, homozygous mice exhibited a trend toward increased pS129 levels in the Triton X-100-soluble fraction, potentially reflecting phosphorylated α-synuclein that is not yet aggregated or is in the early stages of aggregation (*46*–*48*). At 23 months, homozygous mice also exhibited a trend toward increased pS129 levels in the Triton X-100-insoluble [sodium dodecyl sulfate (SDS) soluble] fraction, and the ratio of pS129 to total α-synuclein was increased, consistent with the presence of more mature, potentially pathological aggregates (*49*).

Therefore, the T61I mutation causes an age-dependent increase in α-synuclein aggregation, mirroring elevated pS129 levels observed with PD progression (*50*). This raises the possibility that CHCHD2-induced α-synuclein aggregation may become toxic over time, or at least would if mice lived longer. Moreover, the data indicate that aggregated α-synuclein extends beyond the SNc and striatum, consistent with the possibility that aggregated α-synuclein could also contribute to nonmotor symptoms.

### CHCHD2 mutant mouse brains exhibit a metabolic shift toward glycolysis and the pentose phosphate pathway

The accumulation of aggregated T61I CHCHD2 mutant and CHCHD10 within mitochondria, as well as the disruption of mitochondrial ultrastructure, strongly suggests that mutant T61I CHCHD2 will also disrupt mitochondrial function. Previous studies have highlighted a metabolic switch from mitochondrial respiration to glycolysis as a compensatory mechanism to sustain cellular adenosine triphosphate (ATP) in the context of mitochondrial dysfunction (*51*). Notably, recent work with a DA neuron-specific complex I–deficient mouse model revealed a robust up-regulation of glycolytic genes in DA neurons by RNA sequencing (*52*). Similarly, pronounced elevation of both glycolytic gene mRNA and protein levels were also found in the heart of CHCHD10 point mutant mice (*53*). To determine whether mutant CHCHD2 disrupts energy metabolism, we performed targeted and untargeted metabolomics where mice were infused with [U-^13^C]glucose for 30 min before harvest.

**Fig. 5.**
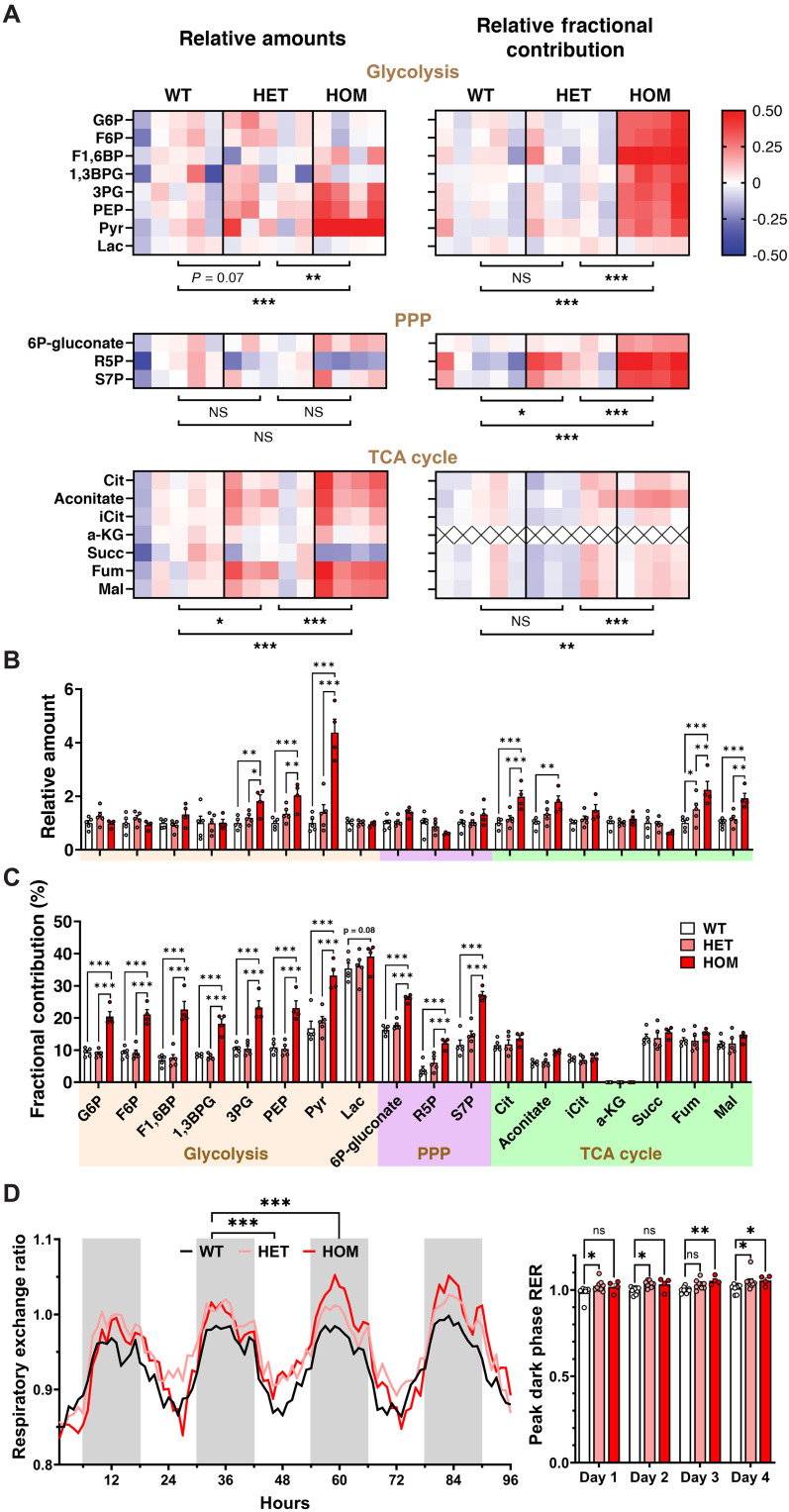
CHCHD2 mutant mouse brains exhibit a metabolic shift toward glycolysis and PPP. (**A**) Female CHCHD2 T61I mice at 19 months received tail vein injections of [U-^13^C]glucose for 30 min before brains were harvested. Brain metabolite extract was analyzed by an ion chromatography-mass spectrometry (IC-MS) detector. Heatmap shows relative total levels of metabolomics in the left column determined by nontargeted metabolomics, and fractional contribution of ^13^C-glucose to each metabolite by targeted metabolomics on the right. Each square shows the log value of a metabolite level or fractional contribution in a mouse normalized to the mean of the WT group. (**B** and **C**) Bar graph of relative amounts (B) or fractional contribution (C) of metabolites along glycolysis, PPP, and TCA cycle. T61I HET and HOM mice showed a dose-dependent increase in relative amounts in distal glycolytic and TCA cycle metabolites (B), and HOM mice showed significantly higher labeling percentages in glycolytic and PPP metabolites. G6P, glucose 6-phosphate; F6P, fructose 6-phosphate; F1,6BP, fructose 1,6-bisphosphate; 1,3BPG, 1,3-bisphosphoglycerate; 3PG, 3-phosphoglycerate; PEP, phosphoenolpyruvate; Pyr, pyruvate; Lac, lactate; R5P, ribose 5-phosphate; S7P, sedoheptulose 7-phosphate; Cit, citrate; iCit, isocitrate; a-KG, α-ketoglutarate; Succ, succinate; Fum, fumarate; Mal, malate. *N* = 5 WT, 5 HET, and 4 HOM T61I female mice at 19 months. (**D**) Respiratory exchange ratio (CO_2_ production to O_2_ consumption), RER, of T61I mice at 5 months was recorded for 5 days. T61I HOM mice showed significant increase in RER in the dark phase. *N* = 9 WT, 9 HET, and 4 HOM. In all panels, data represent mean ± SEM. *P < 0.05, **P < 0.01, ***P < 0.001 by two-way ANOVA with Tukey’s [(A) to (C)] or Dunnett’s (D) post hoc test.

T61I heterozygous and homozygous mice at 19 months exhibited a robust dose-dependent increase in the total level of distal glycolytic metabolites in brain lysates including 3-phosphoglycerate (3PG), phosphoenolpyruvate (PEP), and pyruvate (Pyr) (Fig. 5, A and B). The fractional labeling with ^13^C was also increased across essentially all glycolytic metabolites in homozygous mice versus controls, consistent with an overall increase in brain metabolism of glucose through glycolysis and the pentose phosphate pathway (PPP; Fig. 5, A and C). T61I heterozygous and homozygous mice also showed dose-dependent increases in the fractional labeling of PPP metabolites and in relative amounts of a subset of TCA cycle metabolites. However, fractional labeling of TCA metabolites was not increased in homozygous mice compared to controls, suggesting that their accumulation results from a blockage and reduced turnover, rather than an increased flux of glucose metabolites toward respiration. This overall increase in glucose metabolism through glycolysis and the PPP relative to the TCA cycle is thus likely secondary to a primary disruption in mitochondrial respiration. Moreover, these data indicate that, just as T61I CHCHD2 causes the accumulation of phosphorylated α-synuclein throughout the brain—not only in vulnerable dopaminergic neurons—it also induces a brain-wide metabolic shift.

### CHCHD2 T61I mice show increased carbohydrate consumption

Given our finding of the metabolic shift toward glycolysis, whole-body metabolism of the T61I mice at 5 months was monitored by the comprehensive laboratory animal monitoring system (CLAMS). Throughout the 5 days in CLAMS, both heterozygous and homozygous T61I mice showed an increased respiratory exchange ratio (RER), particularly in the dark phase when mice were more active (Fig. 5D). The decrease in O_2_ consumption per CO_2_ production implies a fuel shift toward carbohydrate consumption for glycolysis without affecting overall energy expenditure as estimated by heat (fig. S4), in line with the disruption in respiration implied by metabolomics. As the measurements of whole-body metabolism primarily reflect changes from tissues outside of the brain, especially skeletal muscle and adipose tissues (*54*), these data show that CHCHD2 T61I mice have systemic metabolic changes that extend beyond the brain.

### Expression of respiratory chain genes is reduced in DA neurons in T61I mice

To investigate how CHCHD2 affects gene expression in DA neurons, we performed spatial genomics (Visium Spatial Gene Expression,10X Genomics) on midbrain sections from 16-month-old CHCHD2 T61I and from 13-month-old CHCHD2 KO mice. Disks demarcating areas of gene expression analysis were selected within the SN and VTA, based on containing at least one entire TH-positive neuron and also expressing characteristic DA genes (DAT, VMAT2, or TH) above a preset threshold level (fig. S5, A and B). We first examined selected pathways, including glycolysis, PPP, respiratory chain, and integrated stress response. Both T61I and CHCHD2 KO led to disruptions in metabolic gene expression (fig. S5, C and D). The glycolytic pathway was down-regulated in the VTA of CHCHD2 KO mice, while unaffected in CHCHD2 T61I mice (fig. S5E). In contrast, expression level of nearly every component in complexes I and III was decreased in the VTA of CHCHD2 T61I mice, but only subtly changed in the KO mice (fig. S5, F and G).

We then performed untargeted analyses to identify individual hits and pathways in these mice. The top 20% of altered genes in either the SN or VTA of the CHCHD2 T61I mice were carefully reviewed (fig. S6A). Unexpectedly, none of these genes was within the top 20% of CHCHD2 KO hits (either SN or VTA), or vice versa (fig. S6B). This suggests that T61I CHCHD2 affects distinct cellular mechanisms from CHCHD2 KO. Among genes with the most pronounced changes in CHCHD2 T61I mice (fig. S6A), we identified three shared hits in both the SN and VTA: *Zfand6*, *Lrmda*, and *Apobec1* (fig. S6C). Zfand6 (zinc finger AN1-type containing 6, also known as AWP1), is a key mediator of tumor necrosis factor–α signaling and negatively regulates nuclear factor κB (*55*–*57*). It was also recently reported to help maintain mitochondrial homeostasis by promoting mitophagy (*58*). Little is known about *Lrmda* (leucine-rich melanocyte differentiation associated, also known as C10orf11), but mutations in this gene cause oculocutaneous albinism (*59*). Apobec1 is a cytidine deaminase enzyme that edits transcripts in various tissues including macrophages and dendritic cells. Deletion of Apobec1 in microglia in mice can exacerbate age-related neurodegeneration (*60*). These genes did not show similar trends in the CHCHD2 KO mice versus their littermate controls. However, the potential roles of these genes in mediating CHCHD2 T61I pathophysiology remain to be determined. In addition, we also performed pathway analysis on the CHCHD2 T61I mice using the gene set enrichment analysis (GSEA) tool, but none passed the false discovery threshold of 0.25.

### Spatial transcriptomics reveals similar changes in patients with PD

To assess whether gene expression changes in the SN and VTA of CHCHD2 T61I mice are replicated in the SNc in sporadic PD, we examined the expression levels of DEGs (differentially expressed genes) in ILB cases versus healthy controls (fig. S7). Because the demarcating disks used in our mouse analysis included both DA neurons and surrounding cells due to the limited resolution of Visium, we performed whole tissue analysis in the human samples without TH^+^ masking. Of the 17 DEGs, only 8 were within our GeoMx human dataset assessing gene expression within SNV TH^+^ cells. Notably, seven of these genes exhibited changes in the same direction as observed in the mouse model, and one (*Cox6a2*) reached significance. These data support the idea that CHCHD2 T61I point mutation may lead to transcriptomic changes similar to those observed in sporadic PD, again supporting the possibility of shared pathophysiologic mechanisms between CHCHD2 T61I and sporadic PD.

### Mitochondrial protein-protein interactions are robustly altered in CHCHD2 T61I mice

**Fig. 6.**
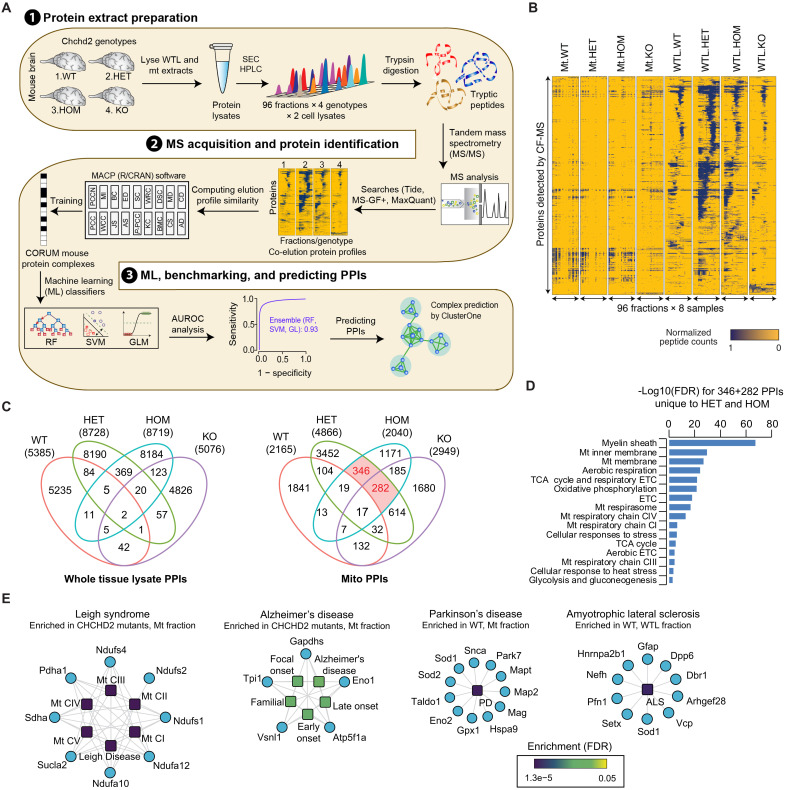
Cofractionation mass spectrometry analysis of protein assemblies across various CHCHD2 genotypes. (**A**) Biochemical fractionation of whole tissue lysates (WTL) and mitochondrial (Mt) lysates from mouse brain with varying *CHCHD2* genotypes was performed using size exclusion chromatography–high-performance liquid chromatography (SEC-HPLC). Protein fractions were subjected to tryptic digestion and analyzed by liquid chromatography with tandem mass spectrometry (LC-MS/MS) to measure peptide spectral counts. The schematic details the computational scoring pipeline from our previous work in Macromolecular Assemblies from Coelution Profiles (MACP). This pipeline includes calculating protein similarity (correlation) metrics for each CHCHD2 genotype, training integrative classifiers with machine learning using the CORUM mouse complex database as a training standard, and scoring cofractionation data to predict high-confidence interactions. These predicted interactions were clustered to define cocomplex membership by the ClusterONE clustering method and analyzed for pathobiological relevance. (**B**) Hierarchical clustering displaying changes in protein cofractionation intensity profiles between Mt. and WTL as measured by LC-MS/MS. (**C**) Venn diagram illustrating the distribution of mouse protein interactions within the Mt. and WTL across different *CHCHD2* genotypes. (**D**) Enrichment analysis of interacting proteins in Mt. mouse brain lysates from *CHCHD2* heterozygous (HET) and homozygous (HOM) samples [red highlights in (C)] involved in annotated metabolic processes. (**E**) Enrichment analysis (FDR ≤ 5 × 10^−2^) of protein assemblies exclusively in different genotypes, either present only in mutants but absent in WT, or vice versa. These assemblies are linked to diseases such as Leigh syndrome, Parkinson’s disease, amyotrophic lateral sclerosis, and Alzheimer’s disease.

While CHCHD2 mutations have been linked to mitochondrial abnormalities, their broader effects on protein assemblies have not been systematically characterized. To address this gap, we applied cofractionation coupled with mass spectrometry (CF-MS) to brains from 15-month-old CHCHD2 T61I HET, HOM, and KO mice. We analyzed 768 fractions across four genotypes (WT, HET, HOM, and KO) using size-exclusion HPLC followed by tandem MS on whole tissue lysates (WTL) and mitochondrial extracts (Fig. 6A). The size exclusion chromatography (SEC) elution profiles showed consistent molecular weights across fractions (fig. S8A). Hierarchical clustering of elution profiles from both WTL and mitochondrial extracts identified 9300 proteins from the mouse proteome in WTL and 297 of 1140 mouse MitoCarta-listed proteins in the mitochondrial extracts (Fig. 6B). This analysis revealed distinct coeluting protein assemblies specific to each extract. Notably, proteins involved in OXPHOS and associated with PD were detected only in mitochondrial extracts and not in WTL (fig. S8B).

Using our recently developed R/CRAN software (*61*), Macromolecular Assemblies from Coelution Profiles (MACP), we calculated correlation scores for each cofractionation experiment. MS2 spectral count profiles across all fractions were generated for each genotype and tissue extract, using three search engines (Tide, MSGF+, and MaxQuant) and compared across 18 elution profile similarity metrics (Fig. 6A). The correlation scores were integrated and tested with multiple machine learning classifiers—random forest, generalized linear model, and support vector machine—and an ensemble model combining all three, to predict protein-protein interactions (PPIs) by fivefold cross-validation against Comprehensive Resource of Mammalian protein complexes (CORUM) mouse protein complexes. The ensemble model showed superior sensitivity over individual models, identifying more CORUM protein pairs without compromising specificity (fig. S8C). Using area under the receiver operating characteristic curve (auROC)-based thresholds (0.79 to 0.94 for WTL and 0.75 to 0.84 for mitochondrial fractions) and filtering out biologically implausible links (i.e., outer membrane–matrix, intermembrane space–matrix), MACP identified 5076 to 8728 PPIs from WTL and 2040 to 4866 from mitochondrial fractions, involving 668 to 1356 and 455 to 818 proteins, respectively (Fig. 6C and fig. S8D). Gene Ontology biological function analysis of the mitochondrial fraction from CHCHD2 mutant–specific intersections (Fig. 6C) showed significant enrichment [false discovery rate (FDR) = 5.0 × 10^−2^] for metabolic processes, including mitochondrial respiration and glycolysis (Fig. 6D). Protein assemblies unique to specific genotypes—present in mutants but absent in WT, or vice versa—were also significantly enriched (FDR ≤ 5 × 10^−2^) for pathways associated with Leigh syndrome, PD, amyotrophic lateral sclerosis (ALS), and Alzheimer’s disease (Fig. 6E).

**Fig. 7.**
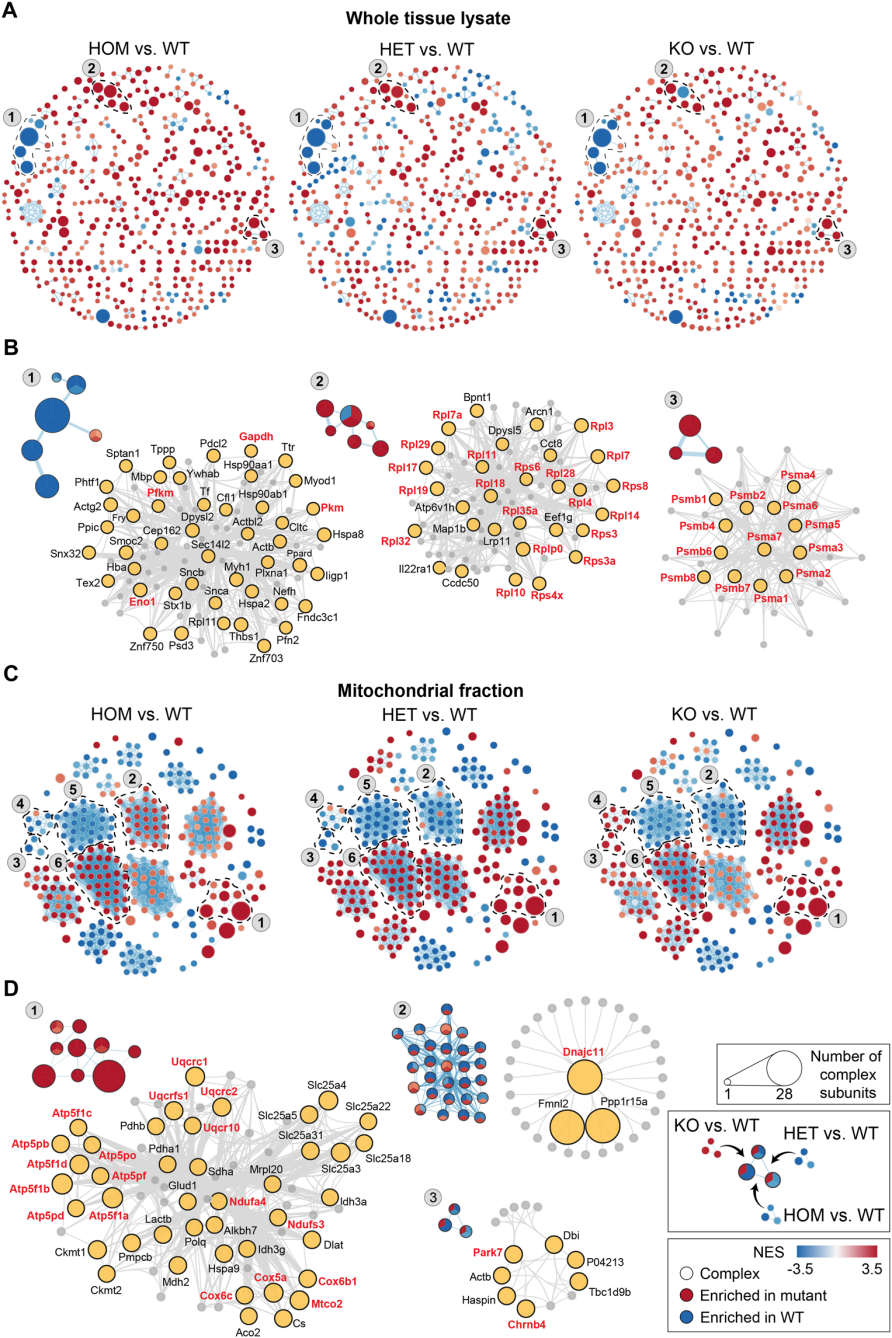
Differential analysis of predicted protein complexes in mouse brain WTL and Mt. lysates across different CHCHD2 genotypes. (**A** and **C**) Complexes in WTLs (A) and Mt. lysates (C) exhibit differential enrichment between CHCHD2 mutants and WT. (**B** and **D**) Displays of representative complexes (indicated by dotted outlines with labels) showing increased interactions in WTLs (B) and Mt. lysates (D) of either CHCHD2 mutants or WT. The left panels in (B) and (D) show the proportion of interactions within each node, detected across various *CHCHD2* genotypes or WT, with node size representing the number of interacting proteins predicted in each complex. The right panels in (B) and (D) provide detailed views of the physically associated proteins within each cluster, with proteins of interest highlighted in red, which are further discussed in the main text.

Following the establishment of the PPI network for each genotype, the ClusterONE (Clustering with Overlapping Neighborhood Expansion) algorithm was used to divide these networks into numerous distinct protein complexes within the mouse brain’s WTL and mitochondrial lysates. The components of these predicted complexes for each genotype were then consolidated into a gene set. This gene set was compared against the coelution profiles to pinpoint differential complexes that showed enrichment in the CHCHD2 mutants or in WT (Fig. 7, A and C). We identified several previously unreported alterations in PPIs, which were shifted either in the same direction or in opposite directions across point mutant and KO genotypes. Changes in the same direction (clusters 1, 5, and 6, mitochondrial fraction) indicate that the T61I mutation exerts impacts similar to the deletion of CHCHD2, implying loss of function. Conversely, changes in the opposite direction (clusters 3 and 4, mitochondrial fraction) reveal additional, previously unrecognized effects of the mutation, supporting a gain of function. Within these complexes, we observed previously undescribed decreases in interactions with glycolytic enzymes (cluster 1) and increases in ribosomal (cluster 2) and proteasomal (cluster 3) interactions in WTL of both CHCHD2 T61I and KO mice (Fig. 7B). Notably, we also identified a significant increase in respiratory chain component interactions in the mitochondrial fraction of both T61I and KO mice (Fig. 7D), underscoring the essential role of CHCHD2 in maintaining respiratory chain assembly. In addition, distinct patterns in T61I homozygous compared to T61I heterozygous and KO mice (cluster 2) highlighted Dnajc11, a cristae-structural protein not previously implicated in CHCHD2 biology, as a potential contributor to neurodegenerative disease. We further uncovered previously unrecognized associations between mutant CHCHD2 and neurodegenerative disease-related genes. These included DJ-1 (Park7) in cluster 3 (Fig. 7D) and cluster 4 (fig. S8E), which causes an autosomal recessive form of PD when mutated. Additional links were observed with Chrnb4 in cluster 3 and Cldn17 in cluster 4, both implicated in frontotemporal dementia (FTD), Nefh in cluster 5, associated with ALS, and Vdac2 in cluster 6, associated with Alzheimer’s disease (fig. S8E).

To validate these altered PPIs, we performed coimmunoprecipitation on 11 selected pairs relevant to mitochondrial metabolism or neurodegeneration. The T61I mutation markedly increased interactions among mitochondrial proteins (fig. S8F) but reduced the interaction between DJ-1 and Chrnb4, which was significantly enriched in CHCHD2 KO (fig. S8G). Together, these results demonstrate substantial disruptions in cellular metabolism caused by CHCHD2 dysfunction and point to previously unidentified links between mitochondrial defects and neurodegeneration.

### CHCHD2 T61I mutation may integrate nonrespiratory chain proteins into supercomplexes

We next investigated how altered mitochondrial PPIs might affect the respiratory chain in the T61I mice by performing blue native PAGE to assess supercomplex assembly at 11 months (fig. S9). While Coomassie blue staining revealed increased supercomplex levels in the mutant mice, a parallel Western blot experiment probing for OXPHOS showed no difference. This discrepancy suggests that the T61I mutation may incorporate proteins beyond the core respiratory chain components into the supercomplexes, in concordance with proteomics profiling.

### CHCHD2 T61I mice have normal complex I activity but progressively elevated reactive oxygen species levels

**Fig. 8.**
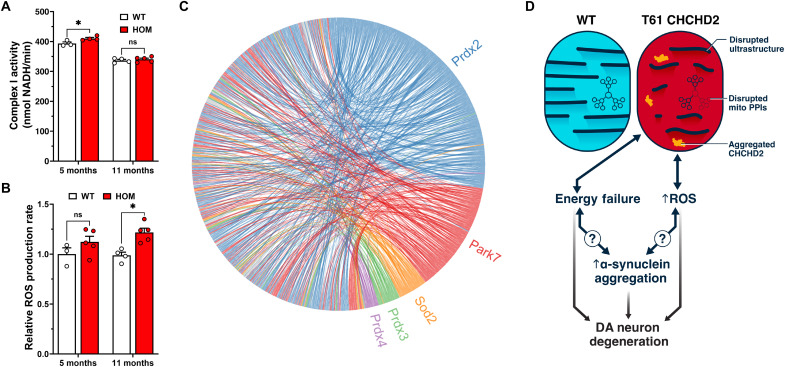
Isolated brain mitochondria show normal complex I activity but progressively rising ROS levels. (**A**) Activity of complex I isolated from brain was slightly increased in T61I HOM mice at 5 months but unchanged from WT at 11 months. (**B**) ROS (H_2_O_2_) levels from brain showed progressive increase in T61I HOM mice from 5 to 11 months. In (A) and (B), *n* = 3 WT and 4 HOM mice at 5 months and 4 WT and 5 HOM mice at 11 months. Data represent mean ± SEM. *P < 0.05 by two-way ANOVA with Sidak’s post hoc test. (**C**) Circos plot showed PPIs with ROS-associated proteins present in the mitochondrial fraction in WT but lost (below threshold) in T61I mutants. Each line represents a PPI between two proteins. Interactions with Prdx2 in blue, Park7 in red, Sod2 in orange, Prdx3 in green, and Prdx4 in purple. (**D**) Proposed pathological model. The T61I mutation leads to CHCHD2 insolubility, likely due to a toxic gain of function, resulting in protein aggregation primarily within mitochondria in SN DA neurons. This mutation also elevates both the total level and phosphorylation of α-synuclein, potentially as a direct consequence or secondary effect of the T61I mutation. Energy failure caused by mitochondrial dysfunction and ROS is proposed to be a key contributor to DA neuron dysfunction and degeneration.

As both spatial transcriptomics and proteomics data suggest a disruption in respiratory chain complex I components, we tested complex I activity by measuring NADH (reduced form of nicotinamide adenine dinucleotide) consumption in isolated brain mitochondria over a 45-min period (Fig. 8A). Mitochondria from 5-month-old female T61I homozygous mice exhibited a slight increase in complex I activity compared to WT mice, with no difference at 11 months. This suggests that respiratory disruption does not result from a specific block at complex I and may instead reflect reduced efficiency across multiple steps in the pathway. Alternatively, respiratory dysfunction may result from dysfunction of mitochondria in specific subpopulations of cells.

In addition to generating ATP, mitochondria are a major source of reactive oxygen species (ROS), and CHCHD2 mutation has been shown to increase oxidative stress in *Drosophila* (*62*) and cultured cells (*43*). To assess mitochondrial ROS in CHCHD2 T61I KI mouse brain, we measured hydrogen peroxide (H_2_O_2_) levels using the Amplex Red assay in isolated brain mitochondria. H_2_O_2_ levels progressively increased between 5 and 11 months of age (Fig. 8B). While no difference was observed at 5 months, T61I homozygous mice showed a significant elevation by 11 months. As this rise in oxidative stress occurs before detectable changes in striatal DA levels or pS129, it may be a key driver of subsequent α-synuclein aggregation and disease progression.

To gain insight into potential mechanisms underlying the increase in ROS, we examined PPIs involving ROS-associated proteins. Notably, all 501 PPIs detected in the mitochondrial fraction of WT mice were lost in T61I mutants, involving 333 proteins. Nearly all of these missing interactions involved PD-associated proteins, including Prdx2 (56.3%), Park7 that encodes DJ-1 (27.5%), Sod2 (6.19%), Prdx3 (5.59%), and Prdx4 (3.59%) (Fig. 8C) (*63*–*70*). This previously unreported collapse of ROS-regulatory interactions reveals a unique disruption of the oxidative stress response network in CHCHD1 T61I mutant mice—an effect not described in prior CHCHD2 models and potentially central to disease pathogenesis.

## DISCUSSION

Our understanding about how mitochondrial dysfunction can cause PD is limited by the lack of robust phenotype in mitochondria-based monogenic mouse models of PD, including Parkin KO, PINK1 KO, and DJ-1 KO (*71*). This may not be unusual given that the mean life span of mice is 26 to 30 months (*72*), while Parkin-, PINK1-, and DJ-1–based PD usually begins after age 20 (*73*). However, both Parkin and PINK1 PD have been reported in patients aged 5 or younger (*74*, *75*), suggesting that other fundamental differences between mice and humans may also contribute to the lack of robust phenotype.

In contrast, even though most patients with T61I CHCHD2-associated PD to date have not presented until their 50s (*28*), and idiopathic PD typically starts after age 50 (*76*), T61I CHCHD2 mice develop profound CHCHD2 aggregation, resembling the aggregation reported in a patient with CHCHD2 PD (*29*). CHCHD2 aggregates were reported to have decreased colocalization with a mitochondrial marker (ATP5A), and hence were interpreted as accumulating in the cytosol, although the percent localizing to the cytosol was not quantified (*29*). Another study also observed decreased colocalization of T61I CHCHD2 with mitochondria in cell lines (*35*). In contrast, in our mouse model, we observe near-complete colocalization of CHCHD2 aggregates with mitochondria, perhaps suggesting that the aggregates first start in the mitochondria, but then a fraction escape to the cytosol. Alternatively, undefined technical or species differences may underlie this discrepancy. We propose that the accumulation of CHCHD2 aggregates leads to the disruption of mitochondrial ultrastructure and mitochondrial PPIs, ultimately leading to mitochondrial dysfunction and a consequent metabolic shift that may be common features by which mitochondrial dysfunction causes different subtypes of both genetic and sporadic PD. In addition to compromising energy metabolism, T61I CHCHD2-induced mitochondrial dysfunction also leads to increased mitochondrial ROS, which may drive the accumulation of aggregated phosphorylated synuclein in midbrain DA neurons, mimicking changes that occur in both CHCHD2-PD (*29*) and idiopathic PD.

### CHCHD2 T61I mouse models have variable phenotypes

Two other CHCHD2 T61I knock-in mouse models have been reported recently, producing somewhat divergent results from ours. The T61I knock-in mouse reported by Torii *et al*. (*35*) demonstrated subtle impairments on rotarod in a very small cohort, and decreased TH fluorescence signal in the SN at 30 weeks, although the study did not directly assess DA neuron degeneration (*35*). In contrast, the other T61I CHCHD2 mouse reported by Fan *et al*. exhibited decreased body weight from 44 weeks, accompanied by a substantial decrease in mouse survival starting at 25 weeks, suggesting undetermined fundamental differences in these mice from our model. The weight loss and decreased mouse survival were accompanied by a roughly one-third decrease in the number of TH-positive neurons in the SN but not VTA at 11 months, although stereology was not used (*36*). Notably, cell loss was not confirmed with another marker, raising the possibility that the decrease in TH neurons reflected down-regulation of TH expression rather than cell death. A substantial loss of TH neurons would be unexpected given the subtle decrease in striatal TH optical density, which suggests little if any loss of terminals (*36*), and would contrast with prior mitochondrial-based models targeting DA neurons where axonal loss precedes neuronal death (*52*). Notably, a third study involving transgenic overexpression of human T61I CHCHD2 in a CHCHD2 WT mouse using a prion promoter (*37*) also reported a substantial decrease in TH cell counts in the SN, with no change in the VTA, as assessed by immunofluorescence, and a subtle decrease in striatal TH intensity. The implications of these findings are difficult to assess, given the potential interactions between exogenous human and endogenous mouse CHCHD2, or the nonphysiological expression pattern driven by the prion promoter. Moreover, methodological details of the quantification were not provided, and it is again unclear whether the observed effects reflect true dopaminergic neuron loss or down-regulation of TH expression.

### Mutant CHCHD2 disrupts mitochondrial respiration and increases ROS

CHCHD2 localizes to the intermembrane space where it associates with the inner mitochondrial membrane (*31*). CHCHD2 and its binding partner CHCHD10 have been shown to support mitochondrial respiration and mediate integrated stress response in different models (*30*–*32*, *77*, *78*). Here, we show that T61I CHCHD2 promotes the preferential accumulation of aggregated CHCHD2 in the mitochondria of DA neurons, and also disrupts their mitochondrial ultrastructure. We hypothesize that this intramitochondrial accumulation of aggregated protein promotes neurodegeneration by both disrupting mitochondrial energy metabolism and increasing mitochondrial ROS production.

Multiple lines of evidence from our mouse model support a broad disruption in mitochondrial energy metabolism. First, analysis of mitochondrial PPIs in brain lysates shows a broad disruption of interactions between respiratory chain proteins in the mouse brain. Second, spatial genomics reveals subtle but widespread decreases in the expression of mitochondrial complex I and III genes in midbrain DA neurons. Third, untargeted metabolomics reveal a total brain accumulation of both TCA and glycolytic metabolites consistent with mitochondrial dysfunction, with increased fractional labeling of glycolytic metabolites again consistent with a glycolytic shift secondary to a primary disruption of mitochondrial respiration. Fourth, whole mouse calorimetry studies reveal proportionally more reliance on carbohydrates relative to fat, also consistent with a deficit in aerobic respiration.

We hypothesize that the aggregation of CHCHD2 plays a causal role in the disruption of respiratory and other mitochondrial PPIs in CHCHD2 mutant mice. However, an alternate possibility is that a primary disruption of mitochondrial PPIs by T61I CHCHD2 leads directly to the disruption of energy metabolism, and the aggregation and accumulation of both T61ICHCHD2 and CHCHD10 are both secondary processes that do not influence the degeneration process, and may even protect against it.

Our metabolomics and whole-animal calorimetry data provide direct evidence of a mutant CHCHD2-induced metabolic shift toward glycolysis in a familial PD model. While a prior study using DA neuron transcriptomics and ex vivo ATP sensors reported a glycolytic shift in the context of a complex I deficiency model of PD (*52*), this study establishes that a similar metabolic shift occurs in a mouse model of familial PD. Our findings demonstrate that mutant CHCHD2, a cause of monogenic mitochondrial PD, drives a glycolytic shift, supporting the idea that this metabolic adaptation may be a shared feature across mitochondrial-associated PD subtypes. Mutant CHCHD2 also strongly decreased D2R-induced somatodendritic hyperpolarization in SNc dopamine neurons. Considering the recently described role of D2Rs in facilitating glycogen storage (*79*), this suggests an additional mechanism by which T61I CHCHD2 may alter glucose metabolism.

It is not yet understood how a glycolytic shift contributes to PD. It may serve as a compensatory mechanism, enabling DA neurons to sustain ATP production as respiratory chain function declines. However, this shift could also be maladaptive, potentially contributing to reciprocal inhibition of residual respiration (*80*, *81*), redox imbalance, aberrant glycosylation, and increased ROS production. Addressing these questions is critical, not only to understand disease pathophysiology, but also because the therapeutic implications differ—it is unclear whether bioenergetic-based therapies should aim to boost glycolysis, restore aerobic respiration, or both.

In addition to disrupting energy metabolism, mitochondrial dysfunction can also increase ROS, which has also been proposed to contribute to PD pathophysiology (*82*). Here, we show that mutant CHCHD2 also increases mitochondrial H_2_O_2_ in isolated brain mitochondria. Moreover, our proteomic analyses revealed robust changes in ROS-associated protein PPIs in the T61I mice, including several PD-associated proteins. In particular, multiple changes in the PD protein DJ-1 were observed, raising the possibility of a mechanistic connection between DJ1 and CHCHD2. DJ-1 was recently reported to directly bind to the CHCHD2 promoter and negatively regulate its expression (*83*). In addition, Kee *et al.* (*37*) also observed a decrease in DJ-1 solubility in the frontal cortex of CHCHD2 T61I mice by unbiased proteomics. CHCHD2 and DJ-1 can function in both the integrated stress response and mitochondrial homeostasis, particularly in response to oxidative stress (*84*–*87*) and may normally act together to maintain mitochondrial quality control. Further investigation is required to determine whether the T61I mutation induces mitochondrial oxidative stress through a DJ-1–dependent mechanism. In addition, both DJ-1 and CHCHD2 were found to interact with F1F0-ATPase. While WT CHCHD2 is suggested to promote F1F0-ATPase assembly, this function is lost with the T61I mutation (*88*). Similarly, DJ-1 directly binds to the β subunit of F1F0 ATPase, and mutation or deletion of DJ-1 increases mitochondrial uncoupling (*89*). Further research will be needed to understand the importance of the CHCHD2 interaction with DJ-1 to normal physiology and PD.

### CHCHD2 mutation causes progressive accumulation of aggregated α-synuclein

Mitochondrial dysfunction and elevated α-synuclein levels independently cause rare monogenic forms of PD and are key features of idiopathic PD, suggesting that either one can cause the other. Supporting this, exposure to the mitochondrial toxin rotenone—a known environmental risk factor for PD—induces ROS and phosphorylated α-synuclein in SH-SY5Y cells (*90*). We show that mutant CHCHD2 leads to an early increase in brain ROS, followed by the accumulation of aggregated α-synuclein, consistent with a model where oxidative stress drives synuclein pathology. Mitochondrial oxidative stress may promote both the aggregation and propagation of α-synuclein (*91*, *92*). Our finding that total body energy disruption also precedes synuclein accumulation raises the possibility that impaired energy metabolism may also independently contribute to aggregation. However, the relative contributions of ROS and energy failure remain difficult to disentangle (*93*).

Our findings in T61I mice, combined with the prominent α-synuclein aggregation observed in a T61I CHCHD2 patient and in T61I CHCHD2-patient-derived IPSCs (*29*), provide compelling evidence that a PD-causing mitochondrial mutation can cause α-synuclein aggregation. This is important in strengthening a causative link between mitochondrial dysfunction and typical later-onset PD, especially because the lack of Lewy pathology in most patients with Parkin-based PD has led many investigators to conclude that autosomal recessive forms of PD are distinct from sporadic PD.

### CHCHD2 accumulation: Driver of SN DA neuron vulnerability or protective mechanism?

Our model system also provides an opportunity to study selective vulnerability. We previously showed that midbrain DA neurons have elevated levels of CHCHD2 relative to several other neuron types (*33*), suggesting a potential mechanism for selective vulnerability. Here, we show that SNc DA neurons preferentially accumulate CHCHD2 aggregates versus VTA DA neurons. We also show that gene expression of CHCHD2 is elevated specifically in vulnerable DA neurons in the ventral tier of the SNc, relative to levels in the dorsal tier. In contrast, this difference was not detected in either presymptomatic cases with ILBs where there is no significant loss of DA neurons or in EPD, suggesting that it is unlikely to reflect the death of DA neurons with higher CHCHD2. Instead, it may reflect a down-regulation of CHCHD2 levels in DA neurons as the disease progresses, which could indicate that it normally protects or is toxic. However, we also observed that CHCHD2 is engulfed in early Lewy aggregates, presumably together with mitochondria (*94*), which are likely degraded as they are no longer evident in more mature Lewy bodies. How this process of mitochondrial degradation influences CHCHD2-specific and overall mitochondrial function is unknown.

In summary, our study positions CHCHD2 as a potentially critical link between primary mitochondrial dysfunction and core pathophysiological mechanisms underlying idiopathic PD, helping to bridge the gap between autosomal dominant and idiopathic forms of PD. Moreover, CHCHD2’s relationship to its binding partner CHCHD10 (*95*, *96*)—implicated in ALS, FTD, and myopathy—supports the hypothesis of shared molecular mechanisms across these disorders, as well as the potential for convergent therapeutic strategies.

## MATERIALS AND METHODS

### Animals

CHCHD2 p.T61I mice (RRID: MGI:7855967) were generated using EUCOMM’s conditional-ready embryonic stem (ES) cells. Two sgRNAs (5′-GCCTCTGAACCTGGCCCATTTGG-3′ and 5′-TCCTGAACCCTATTTAGTTTTGG-3′) respectively targeting immediate upstream of exon 2 and immediate downstream of exon 3 were used to remove the entire exons 2 and 3, and the point mutation was delivered by a donor vector via homologous recombination. ES cells were injected to blastocytes and transferred to recipient females. Mice were maintained on a C57Bl/6 background (The Jackson Laboratory; RRID: IMSR_JAX:000664).

Mice were group housed in a colony maintained with a standard 12-hour light/12-hour dark cycle and given food and water ad libitum. All animal experimental procedures were conducted according to the *Guide for the Care and Use of Laboratory Animals*, as adopted by the National Institutes of Health, and with approval of the University of California, San Francisco, Institutional Animal Care and Use Committee. Animal care and use in this research are covered under the UCSF “Assurance of Compliance with PHS Policy on Humane Care and Use of Laboratory Animals by Awardee Institutions” number A3400-01.

### qPCR genotyping

CHCHD2 p.T61I genotyping was done by qPCR followed by endpoint allelic discrimination. A product size of 322 bp was amplified using the forward primer (5′-TGAAGATGGCCCAGATCTG-3′) and the reverse primer (5′-CTGAAGCCCCCAGTGAT-3′). Allelic discrimination was done automatically by Applied Biosystem’s SDS 2.4 software using the WT probe (5′ JOE-TTAGATGGCTACCACCGCG-3′ Iowa Black) and the MT probe (5′ 6-FAM-TTAGATGGCGATCACCGCG-3′ Iowa Black). A detailed protocol can be found at dx.doi.org/10.17504/protocols.io.kxygxyejwl8j/v1.

### Behavioral testing

General locomotor activity was measured by the open field test using standard procedures (*97*). Mice were placed in the center of a clear acrylic chamber and allowed to explore freely for 15 min. Horizontal and vertical movements were detected using photobeams lining the perimeter of the chamber, and ambulatory and fine movements were separately recorded. Fine movements were defined as breaking the same two photobeams repeatedly, while ambulatory movements were defined as breaking three or more.

Muscle strength was measured by the inverted grid hang test. Each mouse was first placed with all four paws firmly grasping on a wire grid, which was then inverted so the mouse is hanging over a cage filled with bedding. The latency to fall was recorded, and the maximum latency is 180 s. Mice were tested for three trials with an interval of 1 hour between each trial.

Motor coordination was measured using a rotarod (Med Associates Inc.) as described previously (*98*). Fall from the rotating rod was automatically detected by infrared beams, and the maximum latency was 5 min. Five mice of the same gender were tested simultaneously in each trial. On the first day, mice were trained to run on a rotating rod at 16 rpm (training phase). Mice were tested on three trials with an intertrial interval between 15 and 20 min. On the second and third day of testing, mice were placed on the rod rotating at an accelerating speed from 4 to 40 rpm. The rotation speed increased by 7.2 rpm every 60 s. Mice were tested on two sessions of three trials with an intertrial interval between 15 and 20 min, and a 2-hour interval between the AM and PM sessions.

Anxiety-related locomotion was measured using an elevated plus maze (Kinder Scientific) consisting of two open arms and two closed arms elevated 63 cm above the floor. Mice were given at least 1 hour to acclimate to the testing room before freely exploring the maze for 10 min. The time, distance, and entries into the open and closed arms were calculated based on infrared photobeam breaks.

### Electrophysiology

Mice were deeply anesthetized with isoflurane and then decapitated. Horizontal brain slices (150 μm) were prepared using a vibratome (Campden Instruments 7000smz-2). Slices were prepared in ice-cold Ringer solution (119 mM NaCl, 2.5 mM KCl, 1.3 mM MgSO_4_, 1.0 mM NaH_2_PO_4_, 2.5 mM CaCl_2_, 26.2 mM NaHCO_3_, and 11 mM glucose saturated with 95% O_2_/5% CO_2_) and allowed to recover at 33°C for at least 1 hour. Slices were visualized under an Axio Examiner A1 equipped with Dodt and IR optics, using a Zeiss Axiocam 506 mono and Neurolucida (MBF Biosciences) software. Whole-cell recordings were made at 33°C using 4- to 5-megohm pipettes filled with 123 mM K-gluconate, 10 mM Hepes, 0.2 mM EGTA, 8 mM NaCl, 2 mM MgATP, 0.3 mM Na_3_GTP, and 0.1% biocytin (pH 7.2, osmolarity adjusted to 275 mOsm/liter). Recordings were made using Sutter IPA and SutterPatch software (Sutter Instruments), filtered at 5 kHz and collected at 10 kHz. For all current clamp experiments *I* = 0. Liquid junction potentials were not corrected during recordings. The reported firing rates are averages of the spontaneous firing rate over the first 2 min of whole-cell access. *I*_h_ magnitude was measured as the difference between the initial capacitive response to a voltage step from −60 to −120 mV and the final current during the same 800-ms step. Coefficient of variation was calculated over 100 interspike intervals to evaluate the regularity of firing. Input and series resistances were monitored with hyperpolarizing pulses (0.1 Hz) throughout each experiment. All neurons were filled with biocytin during recording, slices were fixed (4% formaldehyde for 2 hours), and then immunocytochemically processed for TH. Agonists, antagonists, salts, ATP, and GTP (guanosine triphosphate) were obtained from MilliporeSigma. Quinpirole effects were statistically evaluated in each neuron by binning data into 30-s data points and comparing the last eight baseline data points to the last eight data points during drug application. All but two neurons recorded in the current clamp were quiescent during quinpirole testing; the two firing neurons were excluded from the figure. Both were from WT mice, and in both cases, quinpirole decreased the spontaneous firing rate. The recorder was blind to genotype. A detailed protocol can be found at dx.doi.org/10.17504/protocols.io.261gedqn7v47/v1.

### Striatal catecholamine analysis

Mice were deeply anesthetized with avertin and perfused with cold phosphate-buffered saline (PBS). Brains were rapidly removed, flash frozen in a dry ice isopentane bath, and stored at −80°C before further processing. The striatal region was dissected from half of a brain cut in the sagittal plane and then trimmed precisely with a cryostat (CryoStar NX50). After the dorsal striatum was fully exposed, punches were made with a pipette tip precut into 1 mm in diameter, and then flash frozen and stored at −80°C. Catecholamine levels were measured by the Vanderbilt Neurochemistry Core by HPLC coupled to an electrochemical detector (*99*). A protocol can be found at dx.doi.org/10.17504/protocols.io.dm6gp97xdvzp/v1.

### Immunohistochemistry and immunofluorescence staining

Mice were deeply anesthetized with avertin and perfused with cold PBS followed by 4% paraformaldehyde (PFA). Brains were dissected out, postfixed in PFA overnight, and stored in 30% sucrose cryoprotectant at 4°C. Brains were cut in the coronal plane into 40-μm-thick sections with a sliding microtome (Leica SM2000R).

For immunofluorescence, brain sections were washed with PBS and transferred in blocking solution containing 0.2% Triton X-100 with 4% bovine serum albumin (BSA) for 1 hour. Sections were then incubated overnight at 4°C with the appropriate primary antibody (Table 1). Sections were then washed with PBS with 0.2% Triton X-100 and incubated for 2 hours at room temperature (RT) with the corresponding secondary antibodies. A detailed protocol for immunofluorescence can be found at dx.doi.org/10.17504/protocols.io.kxygx38owg8j/v1.

**Table 1.** Key resources. Summary of antibodies, critical reagents, experimental models, protocols, software, and scripts used in this study, including source and identifier information for reproducibility.

Materials	Source	Identifier
*Antibodies*		
ABC-HRP kit, peroxidase (Mouse IgG)	Vector Laboratories PK-6102	AB_2336821
ABC-HRP kit, peroxidase (Rabbit IgG)	Vector Laboratories PK-6101	AB_2336820
Aconitase 2 antibody	Proteintech 67509-1-Ig	AB_2882730
Alexa Fluor secondary antibody (Alexa 488 anti-mouse)	Invitrogen A11029	AB_2534088
Alexa Fluor secondary antibody (Alexa 488 anti-mouse)	Invitrogen A21131	AB_2535771
Alexa Fluor secondary antibody (Alexa 488 anti-mouse)	Invitrogen A21121	AB_2535764
Alexa Fluor secondary antibody (Alexa 488 anti-mouse)	Invitrogen A32723	AB_2633275
Alexa Fluor secondary antibody (Alexa 488 anti-rabbit)	Invitrogen A11008	AB_143165
Alexa Fluor secondary antibody (Alexa 555 anti-rabbit)	Invitrogen A32732	AB_2633281
Alexa Fluor secondary antibody (Alexa 568 anti-mouse)	Invitrogen A21124	AB_2535766
Alexa Fluor secondary antibody (Alexa 568 anti-mouse)	Invitrogen A21134	AB_2535773
Alexa Fluor secondary antibody (Alexa 594 anti-rabbit)	Invitrogen A11012	AB_2534079
Alexa Fluor secondary antibody (Alexa 647 anti-mouse)	Invitrogen A31571	AB_162542
Alexa Fluor secondary antibody (Alexa 647 anti-rabbit)	Invitrogen A31753	AB_2536183
Alexa Fluor secondary antibody (Alexa 647 anti-sheep)	Invitrogen A21448	AB_2535865
ATP5A1 antibody	Proteintech 66037-1-Ig	AB_11044196
ATP5C1 antibody	Proteintech 10910-1-AP	AB_2877740
ATPB antibody	Proteintech 17247-1-AP	AB_2061878
CHCHD10 antibody	Proteintech 25671-1-AP	AB_2880187
CHCHD2 antibody	Proteintech 66302-1-Ig	AB_2881685
CHCHD2 antibody	Prestige antibodies HPA027407	AB_10959659
CHCHD2 antibody	Proteintech 19424-1-AP	AB_10638907
CHRNB4 antibody	Proteintech 22192-1-AP	AB_11232227
Citrate synthase antibody	Proteintech 16131-1-AP	AB_1640013
COX6B1 antibody	Proteintech 11425-1-AP	AB_2085449
EM goat/rabbit IgG, ultra small	Electron Microscopy Sciences, 25100	
IRDye 800CW secondary antibody (anti-mouse)	LI-COR 926-32210	AB_621842
IRDye 800CW secondary antibody (anti-rabbit)	LI-COR 926-32211	AB_621843
NDUFS3 antibody	Proteintech 15066-1-AP	AB_2151109
NeuN antibody	MAB 377	AB_2298772
PARK7 antibody	Proteintech 82913-2-RR	AB_3670647
PDH antibody	Abcam ab110333	AB_10862029
PDHA1 antibody	Invitrogen PA5-21536	AB_11152360
PDHB antibody	Proteintech 14744-1-AP	AB_2162941
Phospho-synuclein antibody (pS129)	Abcam ab51253	AB_869973
Phospho-synuclein antibody (pS129)	Abcam ab168381	AB_2728613
TH antibody	BioLegend 818008	AB_2801155
TH antibody	Millipore 152	AB_390204
TH antibody (anti-human)	Santa Cruz Biotechnology sc-374047	AB_10918377
VDAC1 antibody	Abcam ab186321	
Synuclein antibody (Syn-1)	BD Transduction Laboratories 610787	AB_398108
UQCRC1 antibody	Proteintech 21705-1-AP	AB_10734437
UQCRC2 antibody	Proteintech 14742-1-AP	AB_2241442
UQCRFS1 antibody	Abcam ab131152	AB_2716303
*Reagents*		
Amplex Red Hydrogen Peroxide/Peroxidase Assay Kit	Invitrogen A22188	
CFWS gelatin	Aurion 900.033	
Hoechst	Life technologies H1399	
HQ Silver enhancement kit	Nanoprobes 2012	
Kapa Prove Fast qPCR Master Mix (2X) Kit	Kapa Biosystems KK4715	
Visium spatial gene expression slide and reagent kit	10X Genomics 1000184	
[U-^13^C]glucose	Cambridge Isotope Laboratories CLM-1396-1	
*Experimental models*		
C57BL/6J (WT) mouse	The Jackson Laboratory	RRID: IMSR_JAX:000664
CHCHD2 T61I mutant mouse	Self-generated	RRID: MGI:7855967
*Protocols*		
Body composition and metabolic caging analysis in high-fat–fed mice	*Journal of Visualized Experiments*	DOI: 10.3791/57280
CHCHD2 T61I mouse genotyping	Protocols.io	DOI: 10.17504/protocols.io.kxygxyejwl8j/v1
Coimmunoprecipitation	Protocols.io	DOI: 10.17504/protocols.io.bp2l6zxzkgqe/v1
DAB staining of fixed mouse brain tissue sections	Protocols.io	DOI: 10.17504/protocols.io.n92ldm127l5b/v1
Electron microscopy	*Current Protocols in Neuroscience*	DOI: 10.1002/cpns.70
Electrophysiology	Protocols.io	DOI: 10.17504/protocols.io.261gedqn7v47/v1
Human tissue preparation and immunofluorescence staining	Protocols.io	DOI: 10.17504/protocols.io.n92ld8nxxv5b/v2
Immunofluorescence staining of fixed mouse brain tissue sections	Protocols.io	DOI: 10.17504/protocols.io.kxygx38owg8j/v1
In vivo metabolomics	Protocols.io	DOI: 10.17504/protocols.io.kqdg326b7v25/v1
Mitochondrial isolation and complex I activity assay	Protocols.io	DOI: 10.17504/protocols.io.e6nvwdz87lmk/v1
Mitochondrial ROS assay	Protocols.io	DOI: 10.17504/protocols.io.rm7vz9x98gx1/v1
Stereology	Protocols.io	DOI: 10.17504/protocols.io.6qpvr4ekbgmk/v1
Striatal catecholamines analysis	Protocols.io	DOI: 10.17504/protocols.io.dm6gp97xdvzp/v1
Subcellular fractionation	Protocols.io	DOI: 10.17504/protocols.io.4r3l2q4y3l1y/v1
α-Synuclein extraction and Western blot	Star Protocols	DOI: 10.1016/j.xpro.2021.100372
*Software/Equipment*		
10X Space Ranger	10X Genomics	SCR_023571
Applied Biosystems Sequence Detection System software	Applied Biosystems version 2.4	SCR_005039
BaseSpace	Illumina	SCR_011881
Cell Profiler	Broad Institute version 4.2.8	SCR_007358
ClusterONE	Paccanaro Lab	SCR_011848
Cytoscape	Cytoscape v 3.10.1	SCR_003032
Ethovision software	EthoVision version 10	SCR_000441
GeoMx NGS	Salk Institute Next Generation Sequencing Core (NGS)	SCR_014846
GSEA	Broad Institute 4.4.0	SCR_003199
ImageJ/FIJI	National Institute of Health (NIH)	SCR_002285
LIMMA	Walter and Eliza Hall Institute of Medical Research	SCR_010943
Loupe Browser software	Loupe Browser version 5	SCR_018555
MACP	Self-generated	DOI: 10.5281/zenodo.15335295
MaxQuant	Max-Planck-Institute of Biochemistry	SCR_014485
MSGF+	University of California, San Diego	SCR_015646
Neurolucida	MBF Bioscience	SCR_001775
Oxymax-CLAMS	Columbus Instruments	SCR_016718
Percolator	University of Washington	SCR_005040
R	University of Auckland	SCR_001905
ReAdW	Institute for Systems Biology	SCR_025616
Rstudio	Posit, Version 2022.12.0 + 353	SCR_000432
Stereo Investigator	MBF Bioscience	SCR_024705
SutterPatch	Sutter Instrument, Version 2.3.1	
Thermo XCalibur	Thermo Fisher Scientific	SCR_014593
Tide	Netherlands Cancer Institute	SCR_023704
*Scripts/Analysis files*		
CHCHD2 immunofluorescence staining quantification	Self-generated	DOI: 10.5281/zenodo.17307694
Scripts for human spatial transcriptomics analysis	Self-generated	DOI: 10.5281/zenodo.17298932
Scripts for mouse spatial transcriptomics analysis	Self-generated	DOI: 10.5281/zenodo.10819194

For peroxidase staining, sections were quenched with 3% hydrogen peroxide and 10% methanol in PBS. Sections were then blocked in 10% bovine calf serum with 0.4% Triton X-100. Sections were incubated overnight with rabbit anti-TH (1:1000), mouse anti-α-synuclein (1:500) or rabbit anti-phosphorylated α-synuclein (1:300), followed by biotinylated goat anti-rabbit or anti-mouse IgG (1:300) for 2 hours, and then streptavidin-conjugated horseradish peroxidase (HRP) (1:300) for another 2 hours. Staining was visualized by developing in DAB (Sigma-Aldrich D12384) and 0.003% hydrogen peroxide solution for 3 to 5 min. A detailed protocol for peroxidase staining can be found at dx.doi.org/10.17504/protocols.io.n92ldm127l5b/v1.

Brain sections were imaged with a laser scanning confocal microscope (Zeiss LSM880 with Airyscan) or a slide scanner (Leica Aperio Versa). Quantification of fluorescence and area was performed blinded to genotype with ImageJ or CellProfiler.

### Stereology

Total numbers of TH-positive neurons were quantified blinded to genotype. Region selection of SN and VTA was done under 5× magnification following the definition by Fu *et al.* (*100*). Sections were imaged at 63× using a Zeiss Imager microscope (Carl Zeiss Axio Imager A1) equipped with an XYZ computer-controlled motorized stage and an EOS rebel T5i Digital Camera (Canon), and cell counts were performed using MBF Bioscience’s Stereo Investigator. Counting frame size was 60 μm by 60 μm and systematic random sampling (SRS) grid was 130 μm by  130 μm, with a section interval of 6. A protocol can be found at dx.doi.org/10.17504/protocols.io.6qpvr4ekbgmk/v1.

### Quantifications by CellProfiler

Acquired images were first segmented to resolve individual cells, by manually drawing each cell using the Fiji rectangle selection tools. Individual cell image files were then saved in the input folder and batch processed in CellProfiler [v4.2.5]. The parameters and modules of our CellProfiler pipelines used in this study can be found at Zenodo (DOI: 10.5281/zenodo.17307694).

### Subcellular fractionation

Cells were harvested with a scraper, washed in PBS, homogenized using a glass pestle in isolation medium (250 mM sucrose, 1 mM EDTA, and 10 mM tris-Mops, pH 7.4), and then centrifuged at 600*g* for 10 min at 4°C to pellet nuclei and undisrupted cells. The resulting supernatant was centrifuged at 9000*g* for 10 min at 4°C, separating the supernatant (containing cytosol) from the pellet. The pellet was resuspended in isolation medium and subjected to three additional rounds of centrifugation at 9000*g* for 10 min at 4°C, and the final pellet, containing mitochondria, was retained.

For the isolation of soluble (S) and insoluble (I) fractions from the mitochondrial and cytosolic samples, both fractions were incubated on ice with 1% Triton X-100 for 30 min and then centrifuged at 20,000*g* for 10 min at 4°C. After removal of the supernatant (S fraction), the pellet was incubated in SDS buffer for 30 min and centrifuged for 10 min at 20,000*g* yielding the insoluble fraction (I fraction). A detailed protocol can be found at dx.doi.org/10.17504/protocols.io.4r3l2q4y3l1y/v1.

### Electron microscopy

Mice were deeply anesthetized with avertin and perfused with cold PBS followed by 4% PFA and 0.5% glutaraldehyde. Brains were removed and postfixed in the same fixative solution overnight and stored at 4°C. Samples were sectioned into 100-μm thickness with a vibratome. Before immunogold labeling, brain sections were washed three times with 0.1 M phosphate buffer, reduced by 1% sodium borohydride for 30 min, and incubated in 25% sucrose cryoprotectant for 30 min. After four more washes, brain sections were flash frozen with isopentane and freeze-thawed three cycles to increase permeability. Sections were blocked in 0.1 M phosphate buffer with 0.3% bovine serum albumin (BSA, crystallized) and 0.05% sodium azide, incubated overnight at 4°C with the rabbit anti-TH primary antibody (Millipore AB152) and then with 0.8-nm gold particle goat anti-rabbit antibody (EMS, 25100) overnight. Sections were postfixed for 10 min in 1.5% glutaraldehyde solution at RT. After two washes with 0.1 M phosphate buffer and five washes with ddH_2_O, silver enhancement was done for 7 min at RT (Nanoprobes, 2012). A detailed protocol is provided by Zhang and Morales (*101*).

### Postmortem human SN

Tissue samples from pathologically confirmed cases with ILBs, EPD, late PD, and controls without neurological or neuropathological disease were obtained from the Sydney Brain Bank (table S1). The study was approved by the University of Sydney Human Research Ethics Committee (2021/845). All cases with PD were levodopa responsive and fulfilled the UK Brain Bank Clinical Criteria for the clinical diagnosis (*102*) with no other neurodegenerative conditions. Controls had no Lewy pathology, while ILB and PD cases were staged using the regional location of the Lewy pathology (*103*, *104*). Formalin-fixed paraffin-embedded (FFPE) sections from controls, ILB (Braak stages I and II), EPD (Braak stage IV), and late PD (Braak stage VI) were used to examine the expression of *CHCHD2* and *CHCHD10* and the location of their proteins compared with intracellular αSyn aggregations in DA neurons.

### Human tissue preparation and immunofluorescence staining

FFPE blocks of human midbrains containing SN were cut on a rotary microtome (HistoCore MULTICUT R Rotary, Leica Biosystems) at 6 μm. Sections were mounted on Thermo Fisher Scientific (12-550-15) for GeoMx Human Whole Transcriptome Atlas (WTA, nanostring) according to the manufacturer’s instruction or on Series 2 adhesive microscope slides (Trajan Scientific Medical, AU) for immunofluorescence. Before staining, sections were dewaxed and rehydrated with HistoChoice Clearing Agent (Sigma-Aldrich, H2779) and gradient concentrations of ethanol. Each antibody was tested with immunohistochemistry to determine the optimal solution for heat-induced antigen retrieval. For this immunofluorescence approach, formic acid (70% for 20 min) was applied, followed by tris-EDTA (pH 9.0) in a programmable antigen retrieval cooker (Aptum Bio Retriever 2100, Aptum Biologics Ltd., UK). Sections were blocked with Human BD Fc Block (BD Pharmingen, 564219, 1:50) for 30 min at RT and then with TrueBlack Background Suppressor (Biotium, 23012A) for 1 hour at RT. Primary antibodies (either CHCHD2 or CHCHD10 with αSyn and mitochondrial markers) were diluted in blocking buffer (2% donkey serum, 1% BSA, 0.1% fish gelatine, 0.3% Triton X-100, 0.1% Tween-20, and 0.02% NaN_3_ in PBS) for section incubation at RT overnight, followed by the corresponding Alexa Fluor secondary antibodies with Hoechst nucleus counterstaining (bisBenzimide H 33342 trihydrochloride, Sigma-Aldrich, B2261, 1 μg/ml) at RT for 2 hours. Sections were further incubated in Alexa Fluor 790–conjugated TH antibody in blocking buffer at RT for 2 hours before being treated with TrueBlack Lipofuscin Autofluorescence Quencher (Biotium, 23007). Slides were mounted with EverBrite mounting medium (Biotium, 23001) and sealed with Biotium’s CoverGrip Coverslip Sealant (23005). Negative controls were performed for each staining batch, omitting primary or secondary antibodies. A detailed protocol can be found at dx.doi.org/10.17504/protocols.io.n92ld8nxxv5b/v2.

### α-Synuclein extraction and Western blot

Protein was extracted from flash-frozen brains as described by Stojkovska and Mazzulli (*105*). Briefly, homogenized tissues were extracted by agitating in 1% Triton X-100–containing lysis buffer on ice-water slurry for 30 min. Three freeze-thaw cycles were conducted to facilitate homogenization. Lysate was ultracentrifuged at 100,000*g* for 30 min at 4°C, and the supernatant was collected as “Triton X-100-soluble fraction.” The pellet was further extracted with SDS lysis buffer, sonicated, and centrifuged at 100,000*g* for 30 min at 21°C. The supernatant was collected as “SDS-soluble fraction.” Protein concentration was measured using Pierce BCA assay (Thermo Fisher Scientific, 23227).

Fifteen micrograms of total protein from the Triton X-100 fraction and 25 μg from the SDS fraction were loaded onto polyacrylamide gels (Bio-Rad, 4569033) and electrophoresis was carried out using standard SDS-PAGE procedures. Protein was then transferred from the gel onto a polyvinylidene difluoride (PVDF) membrane (Invitrogen, IB24001) using the iBlot 2 rapid transfer system (Invitrogen, IB21001). Following transfer, the remaining gel was stained by Coomassie brilliant blue to serve as a loading control. The PVDF membranes were fixed in 0.4% PFA for 30 min at 20°C before being blocked for 1 hour in a 1:1 mixture of 1× PBS and Li-Cor’s Odyssey PBS blocking buffer (P/N 927-40000). Membrane was incubated overnight with primary antibodies: mouse anti-α-synuclein (Syn-1, BD Transduction Laboratories, 610787, 1:1000), rabbit anti-phosphorylated α-synuclein (pS129 [MJF-R13 (8-8)], Abcam, ab168381, 1:500) and rabbit-anti-Actb (Abcam, ab115777, 1:2500). After incubation in secondary antibodies, Alexa Fluor 488 anti-mouse (Invitrogen, A11029, 1:10,000) and Alexa Fluor 555 anti-rabbit (Invitrogen, A32732, 1:5000), membrane was imaged by Bio-Rad’s ChemiDoc Imaging System. Quantification was done by Bio-Rad’s Image Lab.

### Metabolomics

Mice were fasted overnight before anesthetizing with isoflurane. The mice then received a 200-μl bolus of [^13^C]glucose solution (Cambridge Isotope Labs, CLM-1396-1) via intraperitoneal injection, followed by tail vein infusion at a rate of 150 μl/hour. After infusion, mice were decapitated and the brains were rapidly removed and flash frozen in dry-iced isopentane. Metabolites were extracted from homogenized brains by incubation in 80% methanol at −80°C for 20 min, followed by centrifugation at 14,000*g* for 15 min. Metabolite supernatants were dried in a Labconco CentriVap before storage at −80°C. Samples were quantified at the UCLA Metabolomics Core. A detailed protocol can be found at dx.doi.org/10.17504/protocols.io.kqdg326b7v25/v1.

### Comprehensive laboratory animal monitoring system

Whole-body metabolism was assessed using the CLAMS system, following the standard protocol described by Lancaster and Henstridge (*54*). Briefly, mice were individually housed for 1 week to acclimate before being placed in the CLAMS chambers. They had ad libitum access to their prescribed diet and water and were maintained at 22°C under a 12-hour light/12-hour dark cycle. Food intake, oxygen (O_2_) consumption, and carbon dioxide (CO_2_) production were continuously monitored. The RER was calculated as the ratio of CO_2_ production to O_2_ consumption, while heat production was derived by multiplying RER by O_2_ consumption and an energy equivalent factor, normalized to body weight.

### Spatial transcriptomics

#### Mouse

Spatial transcriptomics were acquired with Visium spatial gene expression kits (10X Genomics). Sample preparation, sample imaging, and library generation were completed in accordance with 10X Spatial Gene Expression protocols. Briefly, fresh brain tissue was flash frozen in a dry ice isopentane bath. The brain tissue was then embedded in Optimal Cutting Temperature compound (Tissue-Tek 62550-12). Sections (10 μm thin) from the midbrain were made with a cryostat (CryoStar NX50) and then mounted on a 10X spatial gene expression slide. Sections were stained with TH, NeuN, and Hoechst 33342 before imaging on a Leica Aperio Versa slide scanner. The cDNA libraries were generated at the Gladstone Genomics Core and sequenced at the UCSF Center for Advanced Technology on an Illumina NovaSeq 600 on an SP flow cell. Alignment of the sequencing data with spatial data from the Visium slide was completed with the 10X Space Ranger software. Two anatomical regions of interest, SN and VTA, and a control region, thalamus, were identified. RNA capture areas corresponding to each anatomical region were selected for analysis based on their spatial proximity to the anatomical regions, and the expression of known genetic markers. SN and VTA genetic markers included TH, DAT, and VMAT2; thalamus markers included Prkcd, Ptpn3, and Synpo2. Demarcation of SN and VTA was done according to Wu *et al.* (*65*). SN and VTA capture areas also had to contain at least one complete DA neuron soma. Gene rankings for hit analysis were established using the fold change score (FCS) and signal-to-noise score (SNS). Equations for these scores are given as

$$\text{SNS}=\frac{\mu_{\text{HOM}}-\;\mu_{\text{WT}}}{\sigma_{\text{HOM}}+\;\sigma_{\text{WT}}}\times \left(-{\text{Log}}_{10}P\right)$$

$$\text{FCS}={\text{Log}}_{2}\left(\frac{\mu_{\text{HOM}}}{\mu_{\text{WT}}}\right)\times \left(-{\text{Log}}_{10}P\right)$$

where μ is the average gene expression, σ is the standard deviation, and *P* is the *P* value derived from a *t* test. Genes with a *P* value <0.05 that also appeared in the top 20% for both scoring metrics were highlighted as DEGs of interest. Code for spatial transcriptomics analysis can be found at Zenodo (DOI: 10.5281/zenodo.10819194).

#### Human

FFPE blocks of postmortem human midbrains were sectioned at 6 μm thickness using a rotary microtome (HistoCore MULTICUT R Rotary, Leica Biosystems). The sections were mounted on Thermo Fisher Scientific slides (catalog no. 12-550-15) and processed using the Nanostring GeoMx Digital Spatial Profiler according to the manufacturer’s instructions, isolating transcripts from either full regions of interest (unmasked) or TH+ (masked) regions (~500 μm). Sequencing libraries were constructed using the Human Whole Transcriptome Atlas (GeoMx Hu WTA) and sequenced on the NovaSeq 6000 following manufacturer protocols. Technical replicates were merged using the Linux “cat” command, and data alignment and feature counting were conducted via the GeoMx NGS analysis pipeline (version 2.0.21) on the Illumina BaseSpace platform. Quality control procedures were executed in R statistical software, applying the following thresholds: minimum segment reads of 1000; ≥80% for percent trimmed, stitched, and aligned; ≥50% for percent saturation; a minimum negative count of 1; a maximum NTC count of 10,000; a minimum of 20 nuclei; and a minimum area of 1000. The overall gene detection rate across all samples was set at a minimum of 1%, with a per-sample minimum gene detection rate also set at 1%. The sequencing of retrieved mRNA probes facilitated spatial transcriptomic measurements of 18,675 transcripts, of which 9012 passed the limit of quantification. Raw counts were subsequently normalized using quantile normalization and DEGs evaluated using LIMMA Voom (*106*). All R scripts used for data processing are available at Zenodo (DOI: 10.5281/zenodo.17298932).

#### Cofractionation coupled with mass spectrometry

WTL and mitochondrial extracts (mt) totaling 500 μg each were obtained from the brains of CHCHD2 WT, CHCHD2 T61 heterozygous and homozygous, and CHCHD2 KO mice. These samples were cross-linked using dithiobis succinimidyl propionate and then subjected to SEC using an Agilent 1100 HPLC system equipped with a 300 mm by 7.8 mm BioSep-4000 Column (Phenomenex), in accordance with a recently described protocol (*61*). This procedure yielded 96 fractions for each extract per genotype. These fractions were subsequently digested with trypsin and analyzed via an Easy-nanoflow liquid chromatography 1200 (Easy nLC; Proxeon) system connected to an Orbitrap Exploris mass spectrometer (Thermo Fisher Scientific). The method for processing the digested samples, the chromatographic separation, and the settings for the comprehensive scanning of mass spectrometry spectra are detailed as previously described (*61*).

#### Liquid chromatography–tandem mass spectrometry

NanoLC connected to an Orbitrap Exploris mass spectrometer (Thermo Fisher Scientific) was used for the analysis of all samples. The peptide separation was carried out using a Proxeon EASY nLC 1200 System (Thermo Fisher Scientific) fitted with a custom-made C18 column (15 cm in length and 150 μm in inner diameter) packed with HxSil C18 3 μm Resin 100 Å (Hamilton). A gradient of water/acetonitrile/0.1% formic acid was used for chromatography. The samples were injected onto the column and run for 95 min at a flow rate of 0.60 μl/min. The peptide separation began with 1% acetonitrile, increasing to 3% in the first 2 min, followed by a linear gradient from 3 to 23% acetonitrile over 74 min, then a steep increase from 24 to 80% acetonitrile over 10 min, and lastly a 10-min wash at 80% acetonitrile. The eluted peptides were ionized using positive nanoelectrospray ionization and directly introduced into the mass spectrometer with an ion source temperature set at 250°C and an ion spray voltage of 2.1 kV. Full-scan MS spectra [mass/charge ratio (*m*/z) 350 to 2000] were captured in Orbitrap Exploris at a resolution of 240,000 (*m*/*z* 400). The automatic gain control was set to 1 × 10^6^ for full FTMS scans and 5 × 10^4^ for MS/MS scans. Ions with intensities above 1500 counts underwent fragmentation in the linear ion trap. The top 15 most intense ions with charge states of ≥2 were sequentially isolated and fragmented using a normalized collision energy of 30%, an activation *Q* of 0.250, and an activation time of 10 ms. Ions selected for MS/MS were excluded from further selection for 30 s. The Orbitrap Exploris mass spectrometer was operated using Thermo XCalibur software. A detailed protocol has been previously described (*61*).

#### LC-MS/MS analysis

Raw spectral files from SEC–high-performance liquid chromatography fractionation-MS experiments were converted into mzXML format using the ReAdW software and then searched against the canonical mouse protein sequences from the UniProt database. To enhance the sensitivity and accuracy of peptide identification, the mzXML files underwent searches against these mouse protein sequences using peptide-spectrum matches from three distinct search engines: Tide (from the Crux suite version 4.1), MS-GF+ (version 2017.01.13), and MaxQuant (version 1.6.7.0) for CF-MS. These searches aimed to maintain an FDR below 0.1% for Tide and MS-GF+ or 1% for MaxQuant, for both peptide and protein identifications. The results from Tide and MS-GF+ were further refined using Percolator. MaxQuant searches were conducted with a 4.5–parts per million (ppm) tolerance for precursor mass in the main search, a 20-ppm tolerance for fragment ion or peptide mass in the first search, and allowance for up to two missed cleavages. Tide searches were set with a 10-ppm tolerance for fragment ion mass and one missed cleavage, while MS-GF+ searches allowed a 20-ppm tolerance for precursor mass with a percolator peptide *Q* value threshold set at 0.1. Across all searches, carbamidomethylation of cysteine was treated as a fixed modification, with methionine oxidation and protein N-terminal acetylation permitted as variable modifications.

#### Computational scoring to predict PPIs from CF-MS datasets

For predicting endogenous PPIs from CF-MS experiments using WTL and mitochondrial (mt) extracts, the MACP R/CRAN software (*61*) was used. The preprocessing steps undertaken included the following: (i) averaging log2-transformed MaxQuant MS1 ion intensities together with spectral counts from Tide and MS-GF+; (ii) excluding proteins identified in only one fraction; (iii) imputing zeros for missing proteins not detected by MS, due to either low abundance or absence in the mouse tissues; and (iv) adjusting for fraction bias and discrepancies in sample injection through row- and column-wise normalization, accounting for the variation in peptide abundance across fractions. Subsequently, the protein elution profile matrix from each search engine for a given sample was independently analyzed for PPI scoring. This analysis used 18 different correlation metrics through the MACP R/CRAN software to calculate pairwise protein profile similarities for each search engine, with details of each profile feature provided in our recent study (*61*). After combining the scores across all search engines, protein pairs were input into three machine learning classifiers (random forest, generalized linear model, and support vector machine), either individually or as a combined ensemble. These models were trained using positive PPIs from CORUM mouse protein complexes, while noninteracting protein pairs from the training set were used as the negative dataset. Model performance was evaluated via fivefold cross-validation using a curated subset of CORUM PPIs held out from training, and area-under-the-receiver-operating-characteristic curve analysis was conducted to assess predictive accuracy. MACP software is available at Zenodo (DOI: 10.5281/zenodo.15335295).

#### Multimeric protein complex predictions from the PPI network

High-confidence PPIs identified through CF-MS experiments across different genotypes were analyzed using the ClusterONE algorithm to delineate complex memberships. The optimization of ClusterONE involved various combinations of parameter adjustments (density, *d*; overlap, *o*) alongside a suite of evaluation metrics (accuracy, maximum matching ratio, and overlap) as we previously described (*61*). In brief, the predicted complexes were compared against known CORUM mouse protein complexes using a range of density (0.2 to 0.45) and overlap (0.5 to 0.8) settings across the evaluation metrics. The parameter settings that yielded the highest composite score—representing the sum of accuracy, overlap, and maximum matching ratio—were deemed most effective for identifying high-quality protein complexes. The interactions among proteins across the four genotypes, along with the predicted macromolecular complexes from the PPI networks derived from CF-MS datasets, were visualized using Cytoscape software (ver. 3.9.1).

#### Differential protein complex analyses

To assess the complex rewiring among the four genotypes, we used coelution profiles of the predicted protein complex subunits. GSEA was performed, treating each complex identified within the four genotypes as a gene set. We then compared the coelution profiles of each subunit within these gene sets across the genotypes.

#### Coimmunoprecipitation

Mitochondria were isolated as described in the “Subcellular fractionation” section. For each immunoprecipitation experiment, as previously described (*107*), 500 μg of mitochondrial lysate, resuspended in 1 ml of RIPA (radioimmunoprecipitation assay) buffer [150 mM NaCl, 50 mM tris-HCl (pH 7.5), 0.1% SDS, 1% Na deoxycholate, 1% NP-40, and 1 mM EDTA] containing protease inhibitor cocktail (PIC), was incubated with 3 μl of antibody at 4°C with agitation for 1 hour. Following this incubation, 100 μl of μMACS Protein A (for rabbit antibodies) and Protein G (for mouse antibodies) magnetic microbeads ( Miltenyi Biotec) was added, and the suspension was further agitated for 4 hours at 4°C. The microbead-bound protein complexes were passed through μMACS columns ( Miltenyi Biotec), washed twice with 1 ml of 0.1% RIPA containing PIC, and then subjected to an additional wash with 1 ml of detergent-free RIPA buffer. Proteins were eluted using 100 μl of 2× Laemmli buffer heated at 95°C, and eluates were analyzed by immunoblotting as previously described (*108*). A detailed protocol can be found at dx.doi.org/10.17504/protocols.io.bp2l6zxzkgqe/v1.

#### Blue native polyacrylamide gel electrophoresis

A detailed protocol for characterizing isolated mitochondria can be found at dx.doi.org/10.17504/protocols.io.e6nvwdz87lmk/v1. A piece of mouse cerebrum containing the midbrain was flash frozen and pulverized for mitochondrial isolation. The mass of isolated mitochondria was measured using a Pierce BCA protein assay kit. To quantify levels of the supercomplex, 50 μg of isolated mitochondria was loaded onto a Native PAGE 3 to 12% gradient gel and stained by Invitrogen’s colloidal blue staining kit (Invitrogen LC6025). Proteins were transferred onto a PVDF membrane by wet transfer, and stained with total OXPHOS antibody cocktail (Abcam ab110411) followed by IRDye 800CW donkey anti-mouse (LI-COR 926-32212). Blots were visualized using a ChemiDoc Imaging System (Bio-Rad 12003153) and analyzed with Bio-Rad’s Image Lab (Bio-Rad v6.1).

#### Complex I activity assay

To measure the enzyme activity of complex I, 10 μg of crude mitochondria preps was dispensed into a 96-well plate containing 50 mM pH 7.4 potassium phosphate buffer, 2 mM potassium cyanide, BSA (2.5 mg/ml), 5 mM MgCl_2_, 2 μM Antimycin A, 100 μM decylubiquinone, and 300 μM K2NADH, with or without 1 μM rotenone. Immediately after combining mitochondria with the above reagents, 340 nm absorbance was measured for 45 min on a Spectramax M4 plate reader. A detailed protocol can be found at dx.doi.org/10.17504/protocols.io.e6nvwdz87lmk/v1.

#### Mitochondrial ROS assay

Mitochondrial hydrogen peroxide (H_2_O_2_) levels were measured using the Amplex Red Hydrogen Peroxide/Peroxidase Assay Kit (Invitrogen, A22188). Crude mitochondrial preps (30 μg) were resuspended in 50 μl of mitochondrial respiration buffer (120 mM KCl, 5 mM KH_2_PO_4_, 10 mM Hepes, 2 mM MgCl_2_, 1 mM EGTA, 5 mM Pyr, and 2.5 mM malate, pH 7.2) and dispensed into a 96-well plate. Following the immediate addition of 50 μl of Amplex Red working solution [100 μM Amplex Red reagent and HRP (0.2 U/ml)], fluorescence was recorded at 590 nm (excitation at 560 nm) for 45 min using a SpectraMax M4 plate reader. A detailed protocol is available at dx.doi.org/10.17504/protocols.io.rm7vz9x98gx1/v1.

#### Quantification and statistical analysis

Details of statistical analyses, including sample size (*n*), its definition, error bar descriptions, statistical tests used, and significance levels, are provided in the figure legends. Linear mixed modeling was applied to compare animal behavioral data. Electrophysiology assessments were analyzed using either a parametric *t* test or a nonparametric permutation test. Two-way analysis of variance (ANOVA) with Sidak’s post hoc test was used for comparisons of catecholamine levels, insoluble CHCHD2 Western blotting, mitochondrial ultrastructure quantification, targeted pathway analysis in Visium spatial transcriptomics, complex I activity assay, and ROS level analysis. Two-way ANOVA with Tukey’s post hoc test was applied to histological analyses, immunofluorescence staining, Western blot quantification, and metabolomics. Human transcriptomic comparisons were conducted using the LIMMA-Voom contrasts of the SNV between CTR and ILB/EPD/LPD with age, sex, and postmortem delay as covariates. CLAMS analysis was assessed using two-way ANOVA with Dunnett’s post hoc test. Differentially expressed genes in Visium spatial transcriptomics were identified by selecting the top 20% of DEGs based on *t* test results. GSEA was used for differential protein complex analysis. Welch’s *t* test was used for blue native polyacrylamide gel electrophoresis supercomplex level comparisons. Seahorse assay data were analyzed using one-way ANOVA with Dunnett’s post hoc test. All statistical analyses were performed using GraphPad Prism (version 10.4.1) and Microsoft Excel.
