# Supplementary material for: CHCHD2 mutant mice link mitochondrial deficits to PD pathophysiology
Source: Sci Adv. 2025 Nov 14;11(46):eadu0726. doi: 10.1126/sciadv.adu0726 (PMC12617525; doi:10.1126/sciadv.adu0726)
Supplement: Supplementary file 1 — Figs. S1 to S9 Table S1 [file sciadv.adu0726_sm.pdf]

Science Advances
AAAS

Supplementary Materials for

CHCHD2 mutant mice link mitochondrial deficits to PD pathophysiology

Szu-Chi Liao *et al.*

Corresponding author: Ken Nakamura, ken.nakamura@gladstone.ucsf.edu; Mohan Babu, mohan.babu@uregina.ca

Sci. Adv. **11**, eadu0726 (2025)
DOI: 10.1126/sciadv.adu0726

This PDF file includes:

Figs. S1 to S9
Table S1

Supplementary Figure 1

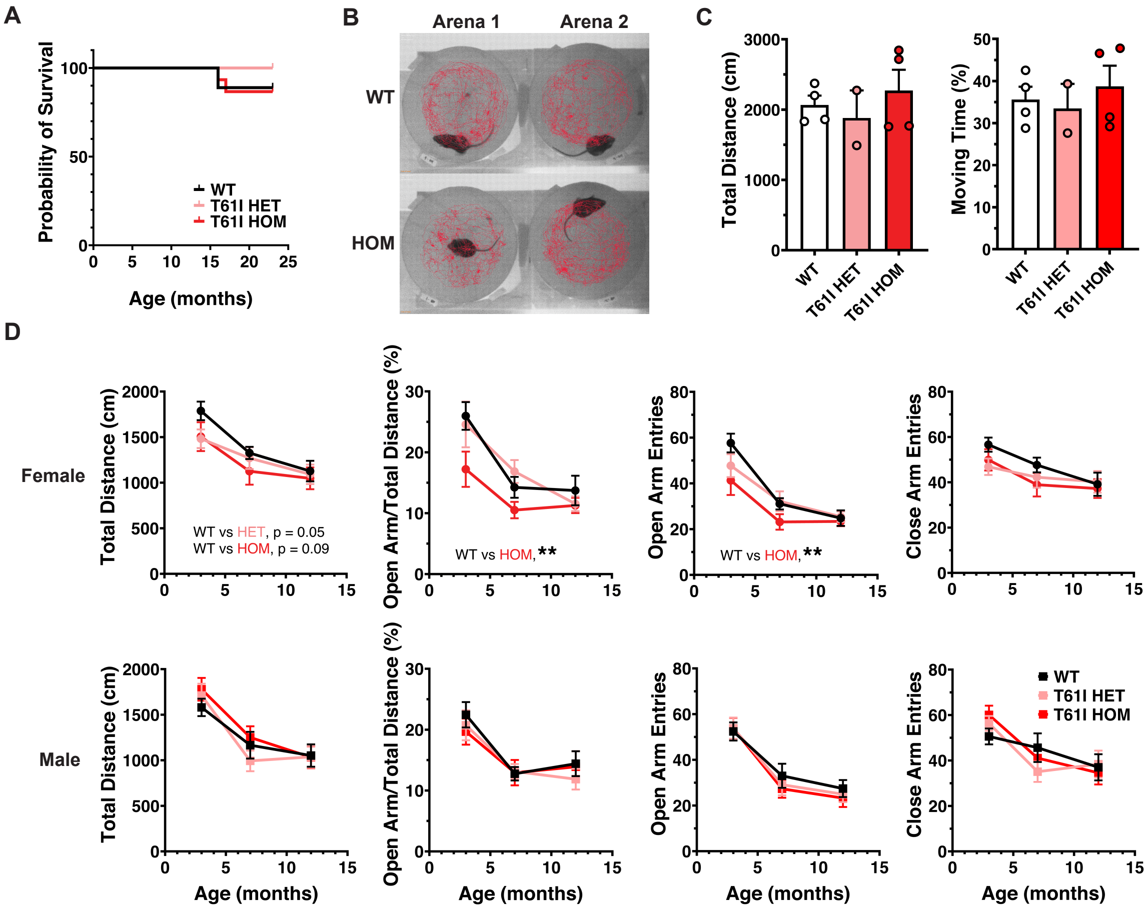

Supplementary Fig. 1: CHCHD2 T61I mice exhibit normal survival and general locomotor activity through 23 months but increased anxiety. (A) Survival probability was not different in a second cohort of T61I mice through 23 months of age (Kaplan-Meier survival curve, n = 9 WT, 8 HET and 15 HOM). **(B)** Representative track visualization of homozygous T61I male mice at 23 months (4 WT, 2 HET and 4 HOM) in the open field test. **(C)** Quantifications of total distance and percentage moving time in the open field test using Ethovision. Data represent mean ± SEM. **(D)** Total movement, open arm/total distance, open arm entries and closed arm entries of T61I mice in the elevated plus maze. Female HET and HOM showed borderline significant (WT vs HET, P = 0.05) and trending (WT vs HOM, P = 0.09) lower total distance, and significantly decreased open arm/total distance percentage (WT vs HOM, P < 0.01) and open arm entries (WT vs HOM, P < 0.01) in the elevated plus maze. N = 16 WT, 16 HET and 13 HOM (female), or 14 WT, 18 HET and 17 HOM (male). Data represent mean ± SEM. **P < 0.01 by linear mixed effects analysis.

Supplementary Figure 2

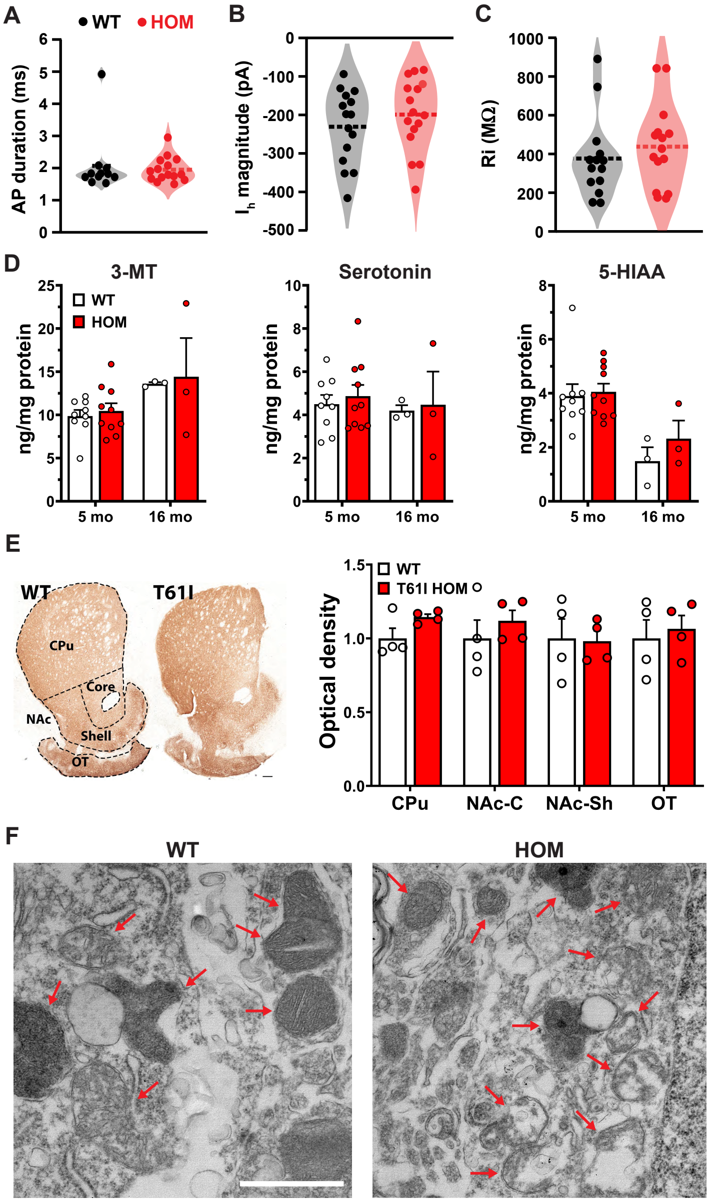

Supplementary Fig. 2: Action potential duration and hyperpolarization-activated current are not altered in CHCHD2 T61I SNc DA neurons. (A) Spontaneous AP waveforms collected in whole cell current clamp configuration. AP duration was measured from threshold to recrossing threshold during repolarization. AP, action potential. **(B)** I_h magnitude was measured in whole cell voltage clamp configuration, $V_{holding}$ = -60 stepping to -120 mV. **(C)** Input resistance was measured in whole cell current clamp mode. N = 5 WT and 4 HOM male mice at 23 months. In (A-C), Each dot is a neuron. Data represent kernel density estimations. For all data parametric assumptions were tested to choose between t-test (parametric) or permutation (non-parametric) analysis. *P < 0.05. **(D)** HPLC of dorsal CPu punches from flash-frozen brain tissues at 5 or 16 months showed no changes in levels of 3-methoxytyramine (3-MT), serotonin, or 5-hydroxyindoleacetic acid (5-HIAA) in T61I HOM mice. N = 9-10 mice/genotype at 5 months and 3 mice/genotype at 16 months. Data represent mean ± SEM. **(E)** Representative images of TH immunoreactivity in striatal sections at 23 months. DA neuron projection areas in dorsal and ventral striatum are indicated with dotted lines. CPu, caudate putamen; NAc, nucleus accumbens; OT, olfactory tubercule. Quantification for TH immunoreactivity showed no difference in WT, HET and HOM by two-way ANOVA with Tukey's *post hoc* test. N = 4 animals/group. Data represent mean ± SEM. Scale bar indicates 300 µm. **(F)** Representative electron microscopy images (quantified in Fig. 3H) demonstrate a higher abundance of swollen mitochondria in SN DA neurons from T61I HOM mice compared with WT. Mitochondria are indicated by red arrows. DA neurons were labeled with a primary TH antibody followed by a gold particle-conjugated secondary antibody. Images were taken at 19,000X magnification, and scale bar indicates 1 µm.

Supplementary Figure 3

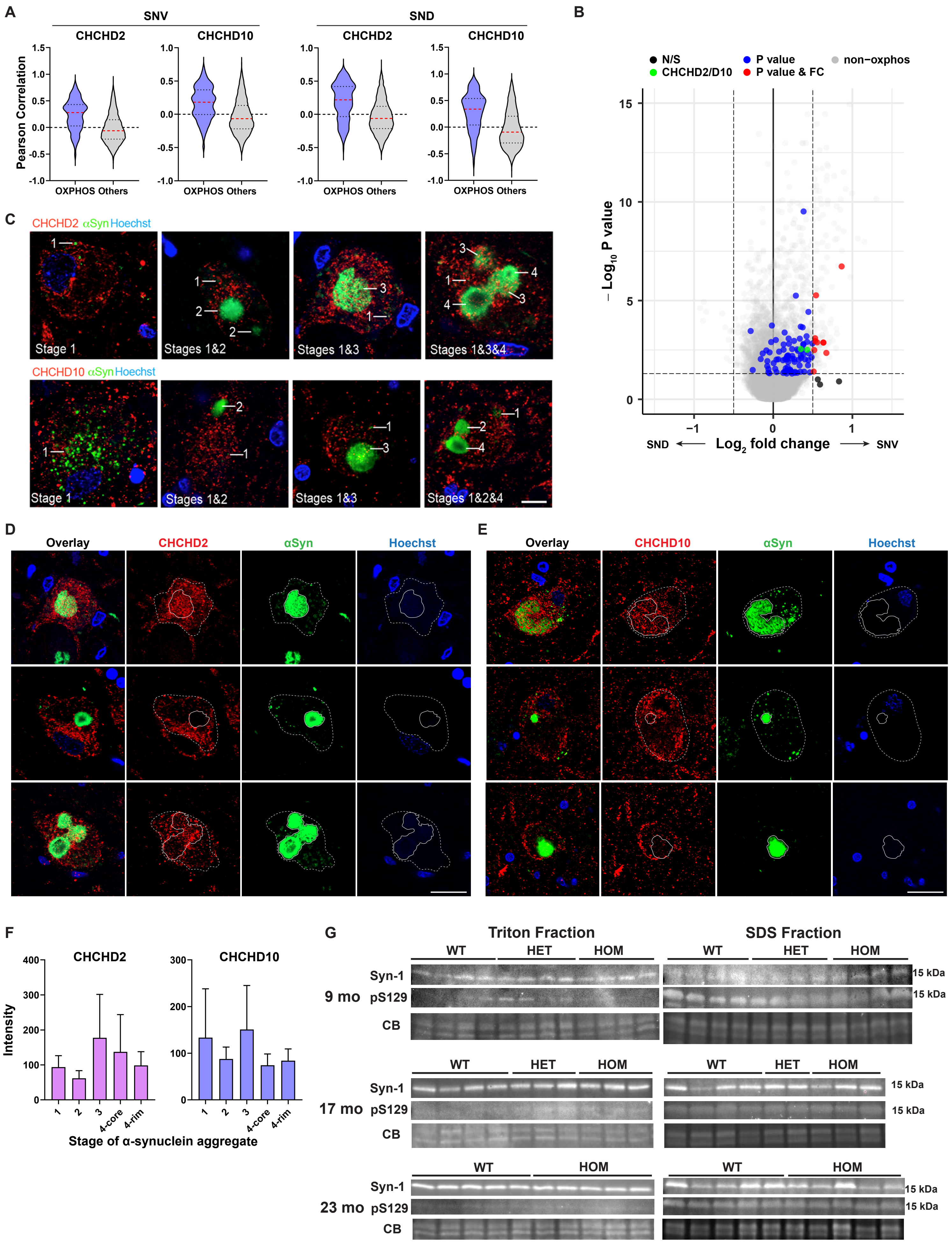

Supplementary Fig. 3: CHCHD2 is incorporated in early-stage Lewy aggregates. (A) Violin plots of Pearson correlation revealed positive correlations between CHCHD2 or CHCHD10 and OXPHOS genes (n = 192) compared with other genes (n = 8,819) assayed by GeoMx. Red dashed line represents median, and black dotted lines represent quartiles. **(B)** Volcano plot showing higher expression of OXPHOS genes in SNV versus SND DA neurons (TH^+ regions of interest). Blue, OXPHOS-related genes surpassing the adjusted P value threshold ($p < 0.05$); red, both P value and log-fold change (FC > 0.5); black, neither P value or FC; green, CHCHD2 and CHCHD10; gray, non-OXPHOS genes. N = 10 control subjects in both (A) and (B). **(C)** Classification of α-synuclein aggregation stage: stage 1, diffuse cytoplasmic α-synuclein; stage 2, small or large cloudy intracellular aggregations; stage 3, net-like intracellular α-synuclein with spots of CHCHD2/10 proteins embedded in pores of the aggregation; and stage 4, ring-shape α-synuclein aggregation with a rim and a core. Scale bar represents 10 µm. **(D and E)** Split channels showing CHCHD2 (D) or CHCHD10 (E) localization in PD SN DA neurons, corresponding to Fig. 4E and F. Scale bars represent 20 µm. **(F)** Quantification of CHCHD2 (left) or CHCHD10 (right) fluorescence intensity within αSyn aggregation at different stages. CHCHD2 or CHCHD10 intensity within aggregation normalized to the sites outside of the aggregates. Data represent mean ± SD. N = 38 Lewy aggregates for CHCHD2 and N = 36 for CHCHD10. **(G)** Gel images of western blot against α-synuclein (Syn-1) and phosphorylated α-synuclein (pS129) in soluble (Triton) and insoluble (SDS) fractions of frontal cortex lysate showed progressive increases from 9 to 23 months. Target protein levels were normalized to total protein content by Coomassie brilliant blue staining. N = 3-5 mice/genotype at each timepoint (except 2 HET in SDS fraction at 17 months).

Supplementary Figure 4

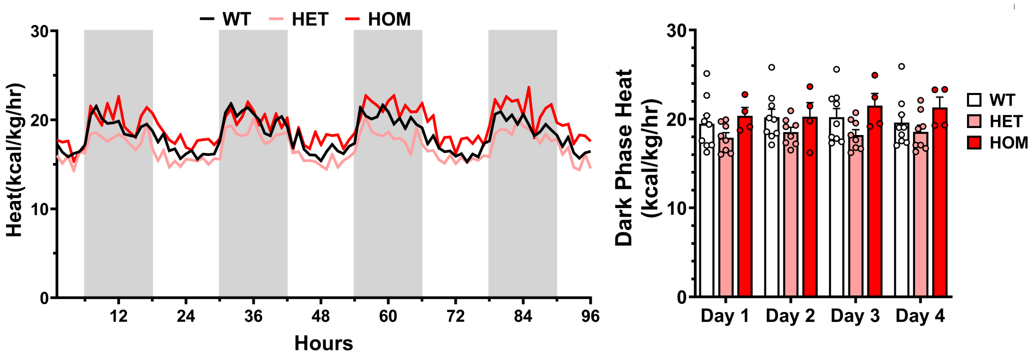

Supplementary Fig. 4: T61I CHCHD2 does not affect whole-body energy expenditure in mice. Whole-time and dark phase energy expenditure as heat were normal in T61I mice. N = 9 WT, 9 HET and 4 HOM mice at 5 months. Data represent mean ± SEM in the bar graph.

Supplementary Figure 5

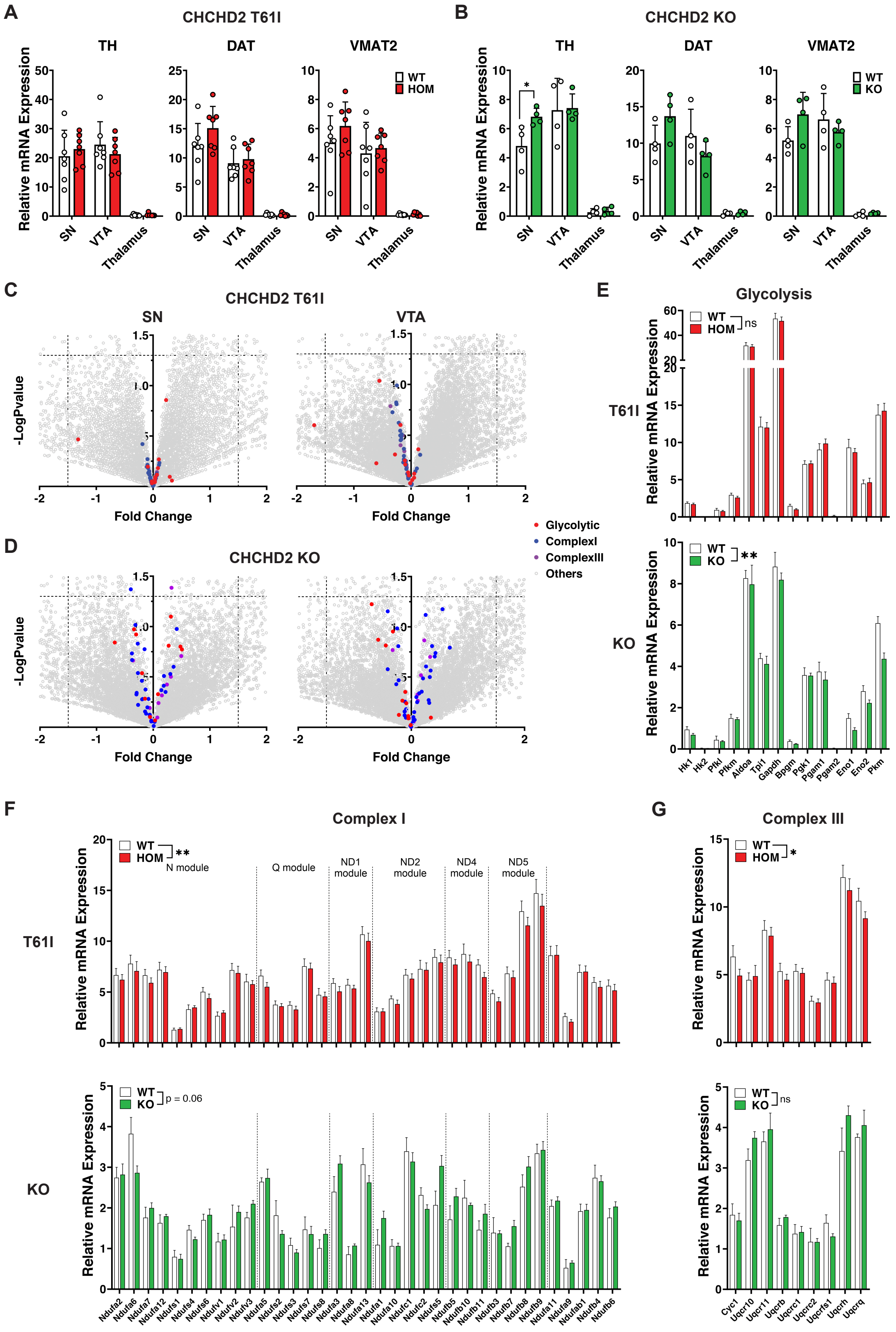

Supplementary Fig. 5: T61I CHCHD2 VTA DA neurons have decreased expression of Complex I and III genes by spatial transcriptomics. Snap-frozen brain sections of CHCHD2 T61I and KO mice were analyzed by the Visium spatial transcriptomics kit. **(A, B)** Expression of DA neuron markers are confined to expected spatial regions. A, CHCHD2 T61I; B, CHCHD2 KO. **(C, D)** Volcano plots showing selected metabolic pathways including glycolytic, complex I and complex III genes in the SN (left) and VTA (right) of CHCHD2 T61I (C) and KO (D) mice. **(E)** Bar graphs of glycolytic gene expression of CHCHD2 T61I (up) and CHCHD2 KO (down) mice. The CHCHD2 KO mice showed a significant downregulation of glycolytic genes compared to their littermate controls. **(F, G)** CHCHD2 T61I mice

exhibited decreased level of nearly all complex I (F) and complex III (G) subunits but not CHCHD2 KO mice. N = 7 homozygous CHCHD2 T61I mice and 7 littermate controls at 16 months and 4 homozygous CHCHD2 KO and 4 littermate controls at 13 months. In all bar graphs (A, B, E-G), data represent mean ± SEM. *P < 0.05, **P < 0.01 by 2-way ANOVA with Sidak's *post hoc* test.

Supplementary Figure 6

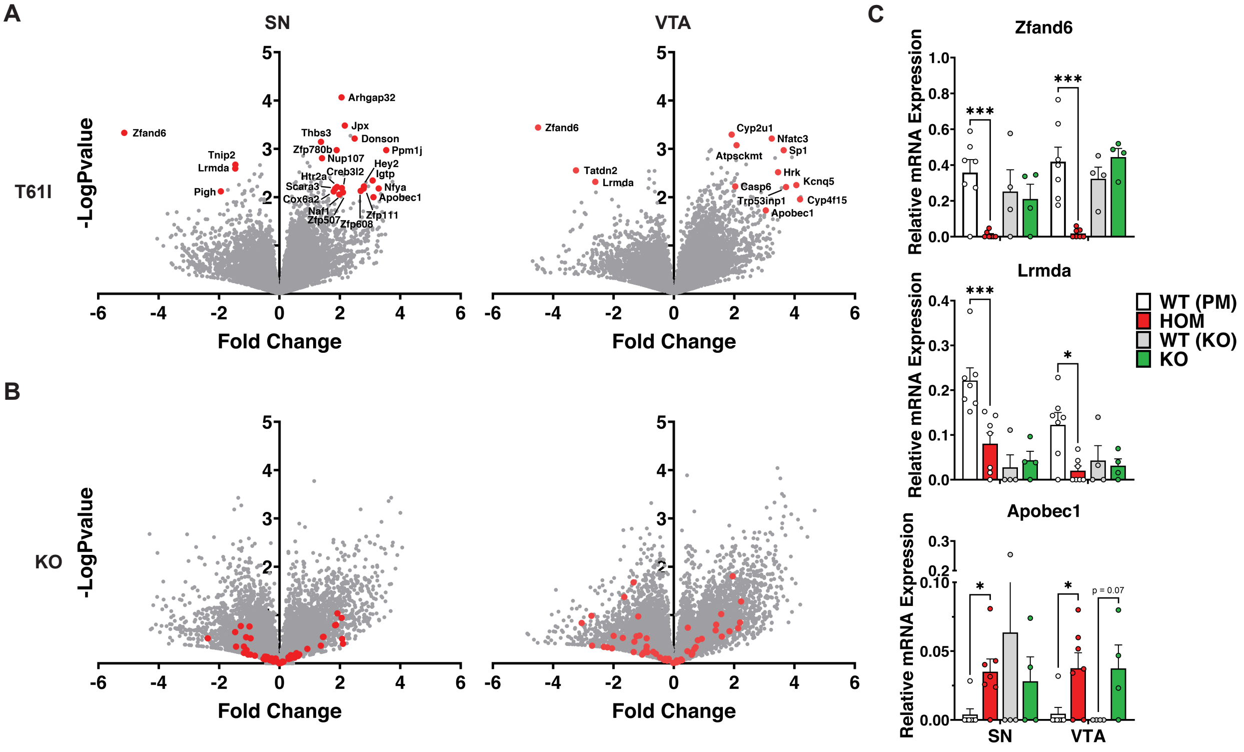

Supplementary Fig. 6: Transcriptomic analysis identifies distinct changes in CHCHD2 T61I and KO mice. Spatial transcriptomics analyses on snap-frozen brain sections of CHCHD2 T61I and KO mice. **(A)** Volcano plots highlighting the top 20 percent individual hits in the SN (left) and VTA (right) of CHCHD2 T61I mice. **(B)** Volcano plots highlighting the individual hits identified from the T61I mice in the SN (left) and VTA (right) of CHCHD2 KO mice. None of the hits was within the top 20 percent in the KO mice. **(C)** Bar graphs of the 3 shared hits in the SN and VTA of the T61I mice, which were unaffected in the CHCHD2 KO mice. N = 7 homozygous CHCHD2 T61I mice and 7 littermate controls at 16 months and 4 homozygous CHCHD2 KO and 4 littermate controls at 13 months. Data represent mean ± SEM. *P < 0.05, ***P < 0.001 by unpaired *t*-test.

Supplementary Figure 7

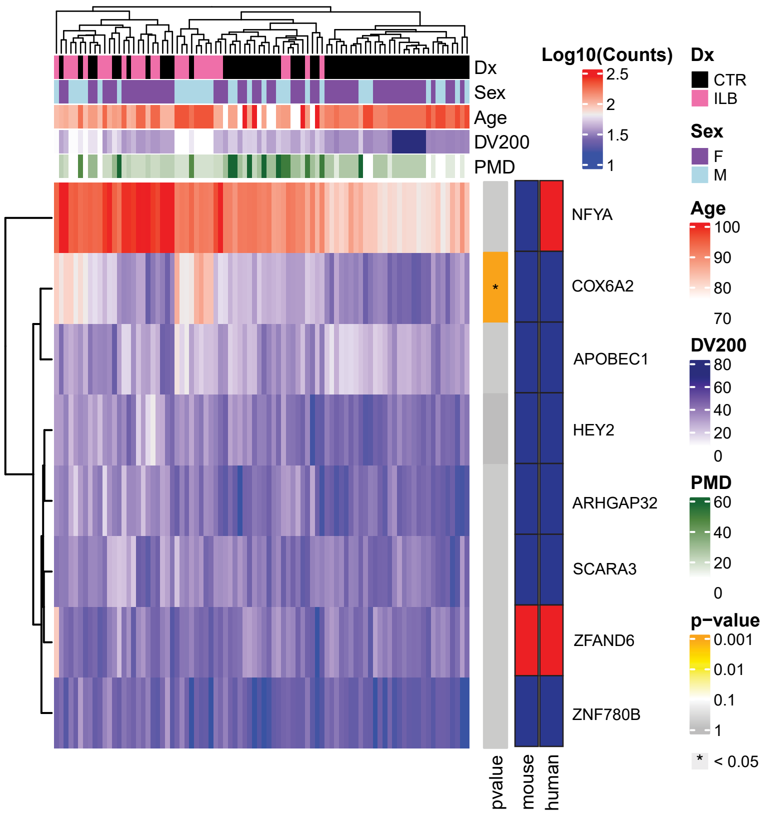

Supplementary Fig. 7: Differentially expressed genes in the CHCHD2 T61I mice are altered in similar trends in cases with Lewy bodies. Heatmap of normalized gene expression (per region of interest) values from GeoMx spatial transcriptomic analysis of human SNc ventral tier within 8 DEG's and identified within the CHCHD2 T61I mouse model. N = 10 (59 regions) controls and 5 (27 regions) incidental Lewy bodies (ILB). 'Limma voom' methodology was used to assess differential gene expression between control and ILB samples. PMD, post-mortem delay; DV200, RNA integrity number equivalent.

Supplementary Figure 8

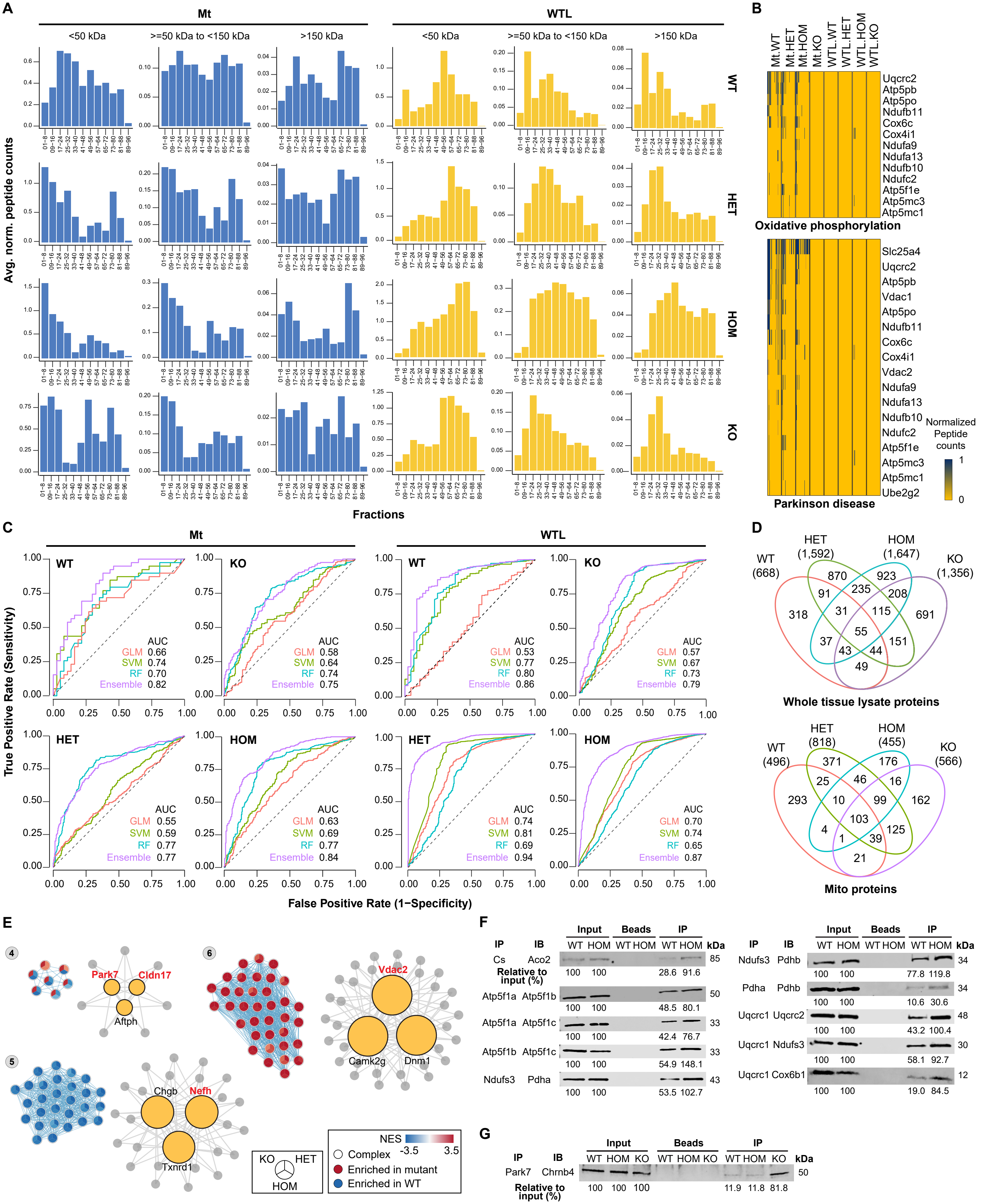

Supplementary Fig. 8: Assessment of the quality of co-eluted proteins detected from CF-MS experiments. (A) Agreement of co-eluting proteins from the mitochondrial (mt) and whole tissue lysate (WTL) with molecular weights (kDa) inferred from SEC profiles. **(B)** Hierarchical clustering of protein co-fractionation, highlighting normalized peptide count profiles recorded by LC-MS/MS with components of OXPHOS and those linked to PD emphasized. **(C)** auROC curves (sensitivity vs. 1 – specificity) showing the performance of ensemble vs. individual machined learning classifiers such as generalized linear model (GLM), support vector machine (SVM) and random forest (RF) evaluated by fivefold cross-validation using the reference CORUM mouse protein complexes. **(D)** Venn diagram showing the overlap of proteins from each genotype corresponding to the PPIs in Fig. 6C, derived from whole tissue lysates and mitochondrial extracts. **(E)** Displays of representative complexes 4 to 6 in Fig. 7C. The left panels show the proportion of interactions within each node, detected across various CHCHD2 genotypes or WT. The right panels provide detailed views of the physically associated proteins within each cluster. **(F and G)** Co-immunoprecipitation (co-IP) confirmed altered protein-protein interactions in CHCHD2 mutant mice. Mitochondrial protein interactions were enriched in T61I HOM mice (F), whereas the CHCHD2 point mutation and knockout differentially affected Park7 and Chrnb4 interaction (G). IB, immunoblotting.

Supplementary Figure 9

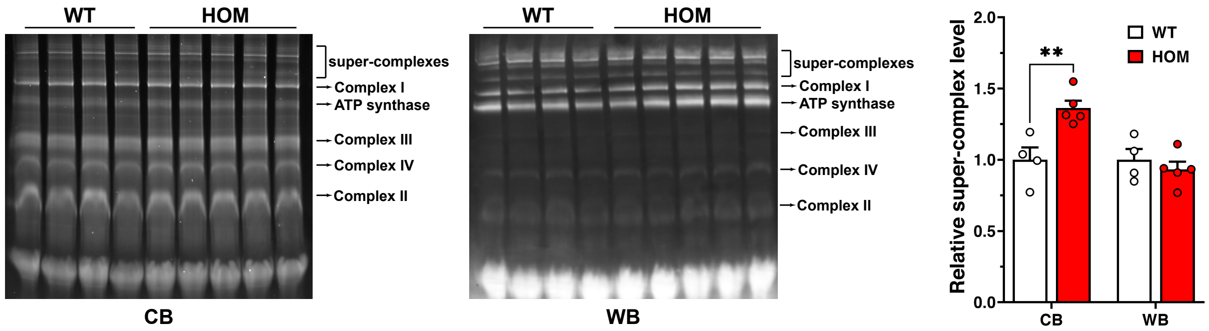

Supplementary Fig. 9: Assessment of Supercomplexes in CHCHD2 T61I mice. Mitochondria were isolated from the brains of 11-month-old mice. Blue native PAGE revealed significantly higher level of supercomplexes by total protein Coomassie blue staining (CB, left panel) but no change by OXPHOS antibody cocktail (WB, center panel). N = 4 WT and 5 HOM mice at 11 months. Data represent mean ± SEM in the bar graph (F). **P < 0.01 by Welch's *t*-test.

Table S1 – Demographics of the human post-mortem cohorts.

	Group	Gender (M/F)	Age at death (years)	Post-mortem delay (hours)
GeoMx WTA	Control	3/7	92.0 (86.25-97.75)	25.0 (16.75-33.25)
	ILB	3/2	88.0 (77.0-93.0)	20.0 (18.0-22.0)
	EPD	5/5	72.0 (63.0-81.0)**	17.5 (3.0-32.0)*
	LPD	5/0	77.0 (67.0-87.0)*	15.0 (12.0-17.0)*
IHC	Control	3/5	77.0 (68.0-78.0)*	15.0 (15.0-17.0)*
	ILB	3/2	93.0 (88.0-96.0)	25.0 (16.8-41.8)
	EPD	2/2	88.0 (85.0-95.0)	20.0 (13.5-35.5)
	LPD	6/1	69.0 (63.8-71.3)*	19.5 (9.8-27.8)

Values are presented as median (Q1-Q3). For the GeoMx WTA cohort, the comparison between gender was performed using χ^2 and a univariate test (Welch Two Sample) followed by Bonferroni correction was used to compare age at death and post-mortem delay. Tests were performed between the Control and each ILB, EPD and LPD group. For the IHC cohort, the comparison of age at death among all groups was made with the Kruskal-Wallis test, followed by Dunn's multiple comparisons. The chi-square test was used for gender differences. ILB = incidental Lewy body; EPD = PD cases with early Lewy pathology (Braak stage 4); LPD =PD cases with late Lewy pathology (Braak stage 6). *P < 0.05, **P < 0.01 compared to controls.
